# Supplementary material for: The Antitumoral Effect In Ovo of a New Inclusion Complex from Dimethoxycurcumin with Magnesium and Beta-Cyclodextrin
Source: Int J Mol Sci. 2024 Apr 16;25(8):4380. doi: 10.3390/ijms25084380 (PMC11050057; doi:10.3390/ijms25084380)
Supplement: Supplementary file 1 [file ijms-25-04380-s001.zip › ijms-2937633-supplementary.pdf]

# The Antitumoral Effect In Ovo of a New Inclusion Complex from Dimethoxycurcumin with Magnesium and Beta-Cyclodextrin

**Marco A. Obregón-Mendoza [1,†], William Meza-Morales [1,†], Karla Daniela Rodríguez-Hernández [1], M. Mirian Estévez-Carmona [2], Leidys L. Pérez-González [1], Rosario Tavera-Hernández [1], María Teresa Ramírez-Apan [1], David Barrera-Hernández [3], Mitzi García-Olivares [3], Brian Monroy-Torres [1], Antonio Nieto-Camacho [1], María Isabel Chávez [1], Rubén Sánchez-Obregón [1] and Raúl G. Enríquez [1,*]**

[1] Instituto de Química, Universidad Nacional Autónoma de México, Mexico City 04510, Mexico; marco.obregon@zaragoza.unam.mx (M.A.O.-M.); william.meza@upr.edu (W.M.-M.); xandy411@comunidad.unam.mx (K.D.R.-H.); leidyslaura92@gmail.com (L.L.P.-G.); rosario.tavera@gmail.com (R.T.-H.); mtrapan@yahoo.com.mx (M.T.R.-A.); brianuami@gmail.com (B.M.-T.); camanico2015@yahoo.com (A.N.-C.); isabel@unam.mx (M.I.C.); rubens@unam.mx (R.S.-O.)

[2] Departamento de Farmacia, Escuela Nacional de Ciencias Biológicas, Instituto Politécnico Nacional, M. Wilfrido Massieu SN, U. A. Zacatenco, Mexico City 07738, Mexico; mirianestevezc@gmail.com

[3] Departamento de Biología de la Reproducción "Dr. Carlos Gual Castro", Instituto Nacional de Ciencias Médicas y Nutrición Salvador Zubirán, México, Mexico City 14080, Mexico; barrera1912@gmail.com (D.B.-H.); mariol_48@ciencias.unam.mx (M.G.-O.)

* Correspondence: habib@unam.mx or enriquezhabib@gmail.com; Tel.: +52-55562-24404

† These authors contributed equally to this work.

Index

**Fig S1.** $^{1}H$ NMR spectrum of Dimethoxycurcumin-$BF_2$ ( DiMeOC-$BF_2$, 400 MHz, DMSO-$d_6$). 3
**Fig S2.** $^{13}C$ NMR spectrum of Dimethoxycurcumin-$BF_2$ ( DiMeOC-$BF_2$, 100 MHz, DMSO-$d_6$). 4
**Fig S3.** IR Spectrum of DiMeOC-$BF_2$. 5
**Fig S4.** Mass Spectrum of DiMeOC-$BF_2$ ($IE^+$). 6
**Fig S5.** 1H NMR spectrum of DiMeOC (400 MHz, DMSO-$d_6$). 7
**Fig S6.** 13C NMR spectrum of DiMeOC (100 MHz, DMSO-$d_6$). 8
**Fig S7.** UV-VIS spectrum in methanol of DiMeOC. 9
**Fig S8.** HPLC spectrum of DiMeOC (417nm, $CH_3CN/H_2O$ (0.02% formic acid) 55:45). 10
**Fig S9.** Mass Spectrum of DiMeOC ($DART^+$). 11
**Fig S10**. $^{1}H$ NMR spectrum of DiMeOC-Mg (400 MHz, DMSO-$d_6$). 12
**Fig S11.** $^{13}C$ NMR spectrum of DiMeOC-Mg (100 MHz, DMSO-$d_6$). 13
**Fig S12.** HSQC NMR spectrum of DiMeOC-Mg (400 MHz, DMSO-$d_6$). 14
**Fig S13.** HMBC NMR spectrum of DiMeOC-Mg (400 MHz, DMSO-$d_6$). 15
**Fig S14.** IR Spectrum of DiMeOC-Mg. 16
**Fig S15.** UV-VIS spectrum in methanol of DiMeOC-Mg . 17
**Fig S16.** HPLC spectrum of DiMeOC-Mg (417 nm, $CH_3CN/H_2O$ (0.02% formic acid) 55:45). 18
**Fig S17.** Mass Spectrum of DiMeOC-Mg (MALDI-TOF). 19
**Fig S18**. $^{1}H$ NMR spectrum of DiMeOC-Mg-BCD (400 MHZ, DMSO-$d_6$). 20
**Fig S19.** $^{13}C$ NMR spectrum of DiMeOC-Mg-BCD (100 MHz, DMSO-$d_6$). 21
**Fig S20.** HSQC NMR spectrum of DiMeOC-Mg-BCD (400 MHz, DMSO-$d_6$). 22
**Fig S21.** HMBC NMR spectrum of DiMeOC-Mg-BCD (400 MHz, DMSO-$d_6$). 23
**Fig S22.** IR Spectrum of DiMeOC-Mg-BCD. 24
**Fig S23.** UV-VIS spectrum in methanol of DiMeOC-Mg-BCD . 25
**Fig S24.** HPLC spectrum of DiMeOC-Mg-BCD (417nm, $CH_3CN/H_2O$ (0.02% formic acid) 55:45). 26
**Fig S25.** Mass Spectrum of DiMeOC-Mg-BCD (ESI). 27
**Fig S26.** Standard curve of DiMeOC-Mg (415nm, 1-Octanol). 28
**Fig S27.** Inclusion ratio DiMeOC-Mg and BCD (PBS media). 29
**Fig S28.** 1H NMR spectrum of Beta-cyclodextrin (BCD, 400 MHz, DMSO-$d_6$). 30
**Fig S29.** $^{13}C$ NMR spectrum of BCD (100 MHz, DMSO-$d_6$). 31
**Fig S30.** HSQC NMR spectrum of BCD (500 MHz, DMSO-$d_6$). 32
**Fig S31.** HMBC NMR spectrum of BCD (500 MHz, DMSO-$d_6$). 33
**Fig S32.** IR Spectrum of BCD. 34

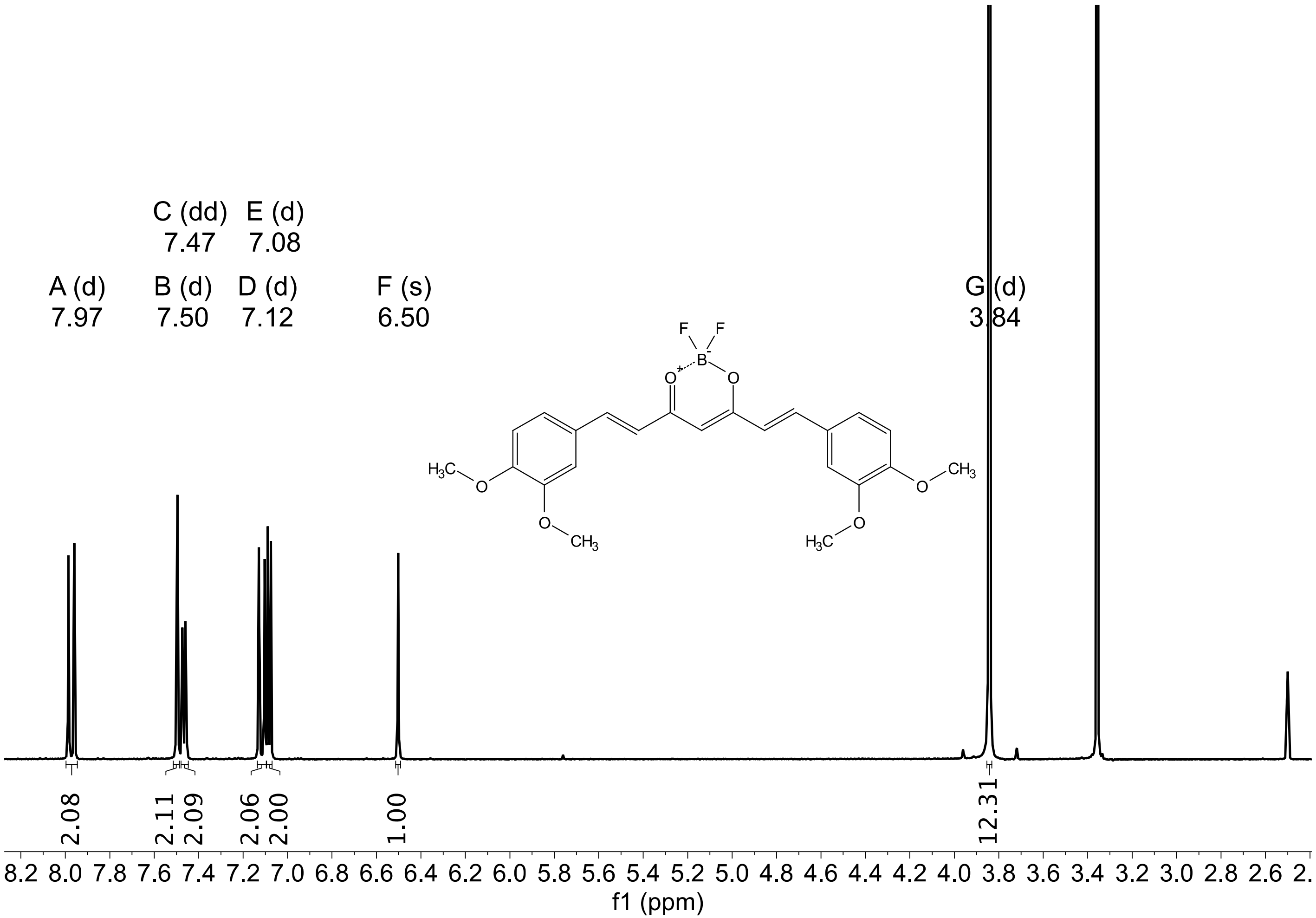


**Fig S1.** $^{1}$H NMR spectrum of Dimethoxycurcumin-BF$_2$ ( DiMeOC-BF$_2$, 400 MHz, DMSO-$d_6$).

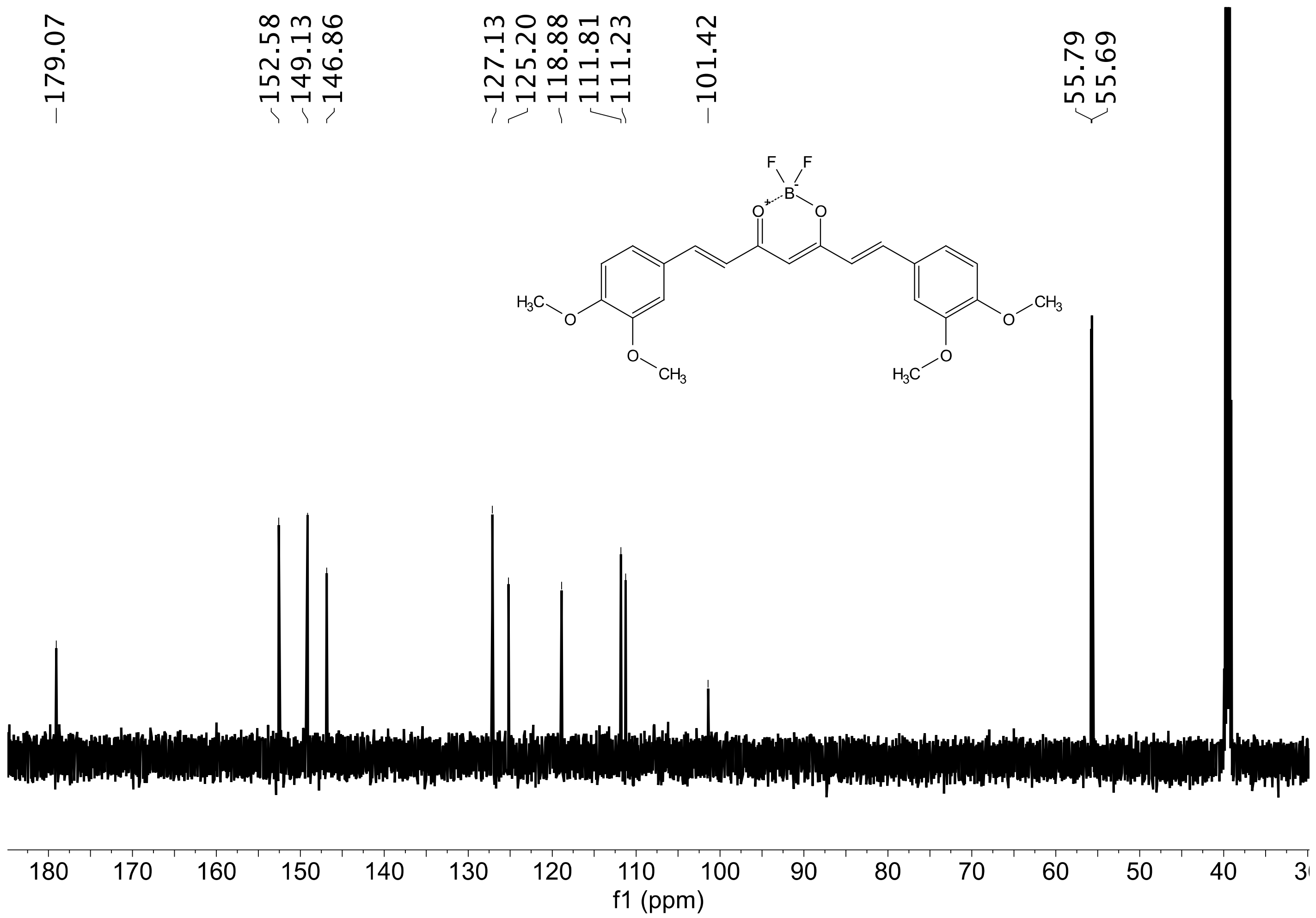


**Fig S2.** $^{13}$C NMR spectrum of Dimethoxycurcumin-BF2 ( DiMeOC-BF2, 100 MHz, DMSO-*d*6).

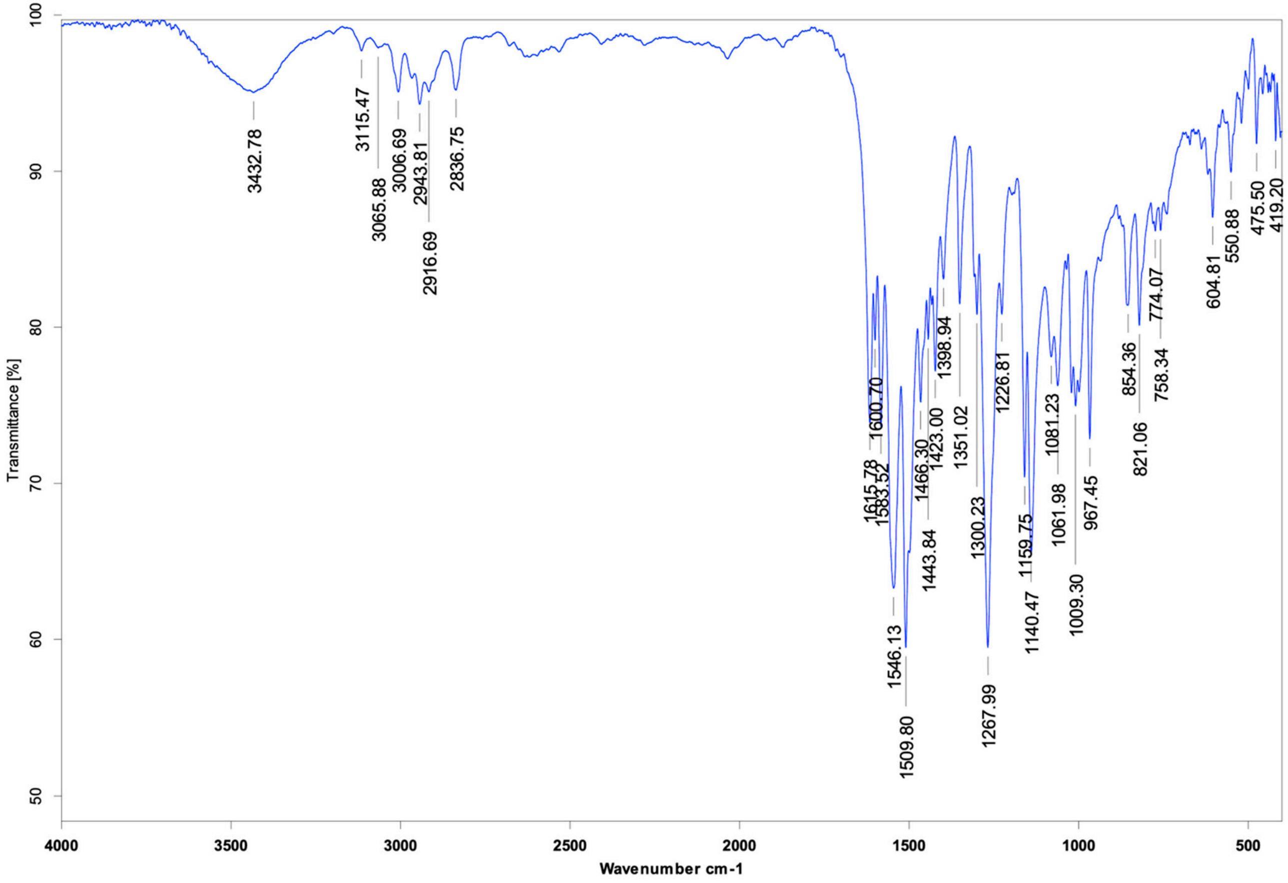


**Fig S3.** IR Spectrum of DiMeOC-$BF_2$.

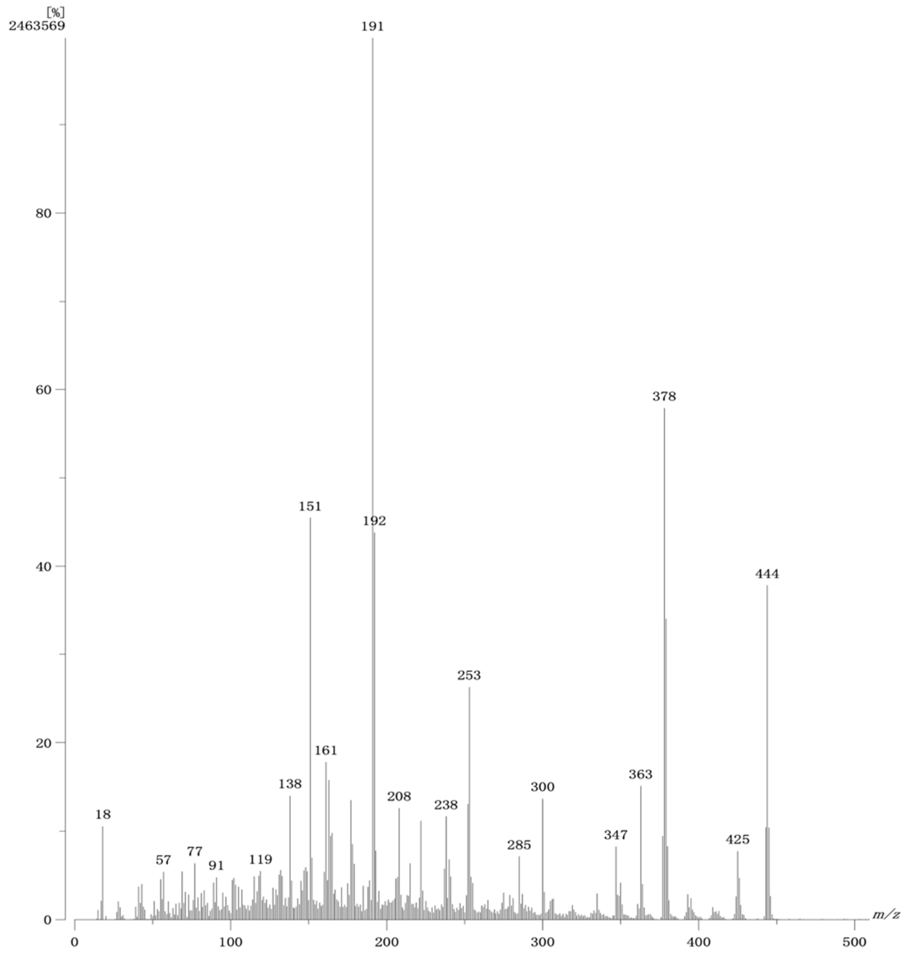


**Fig S4.** Mass Spectrum of DiMeOC-$BF_2$ ($IE^+$).

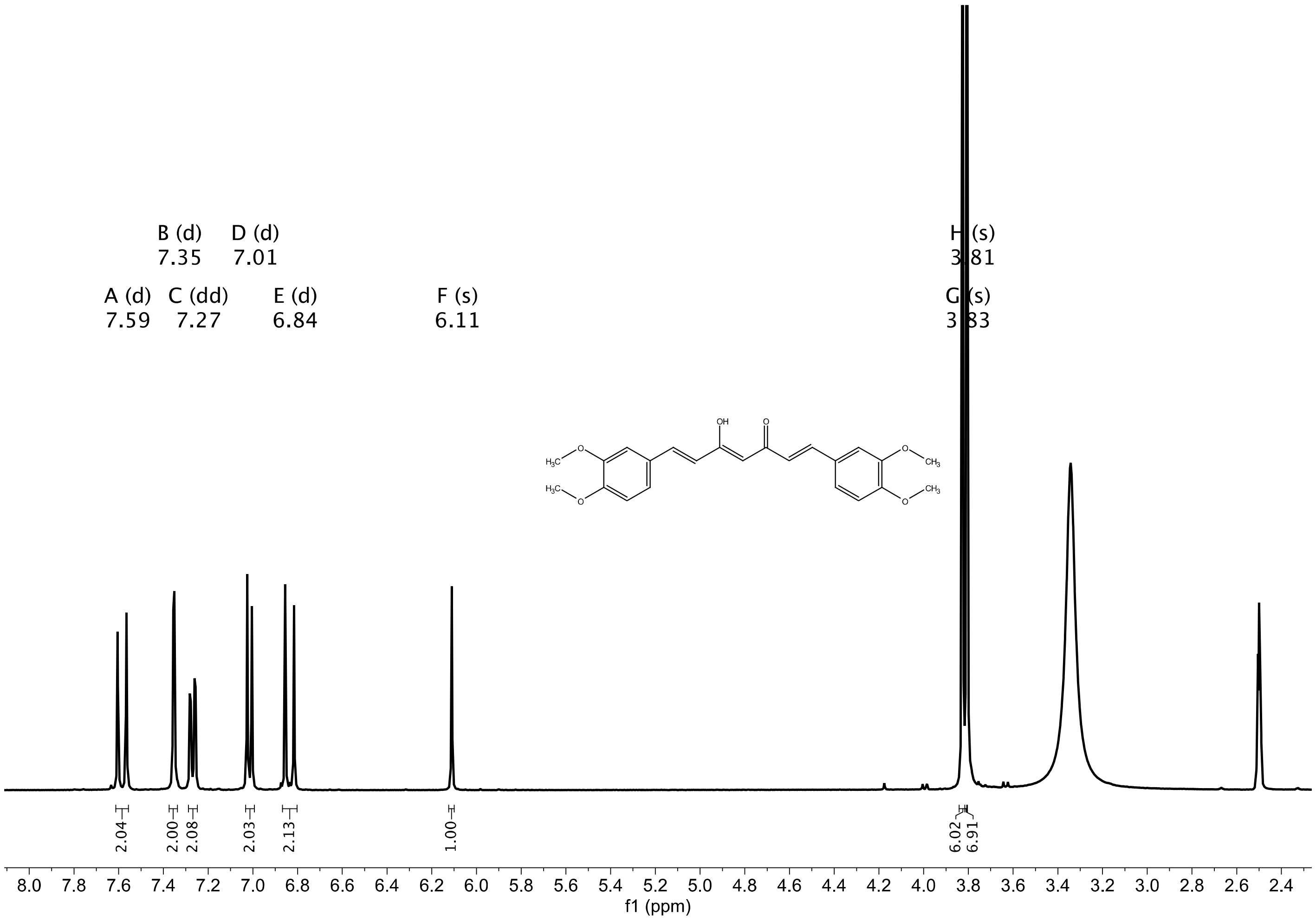


**Fig S5.** 1H NMR spectrum of DiMeOC (400 MHz, DMSO-$d_6$).

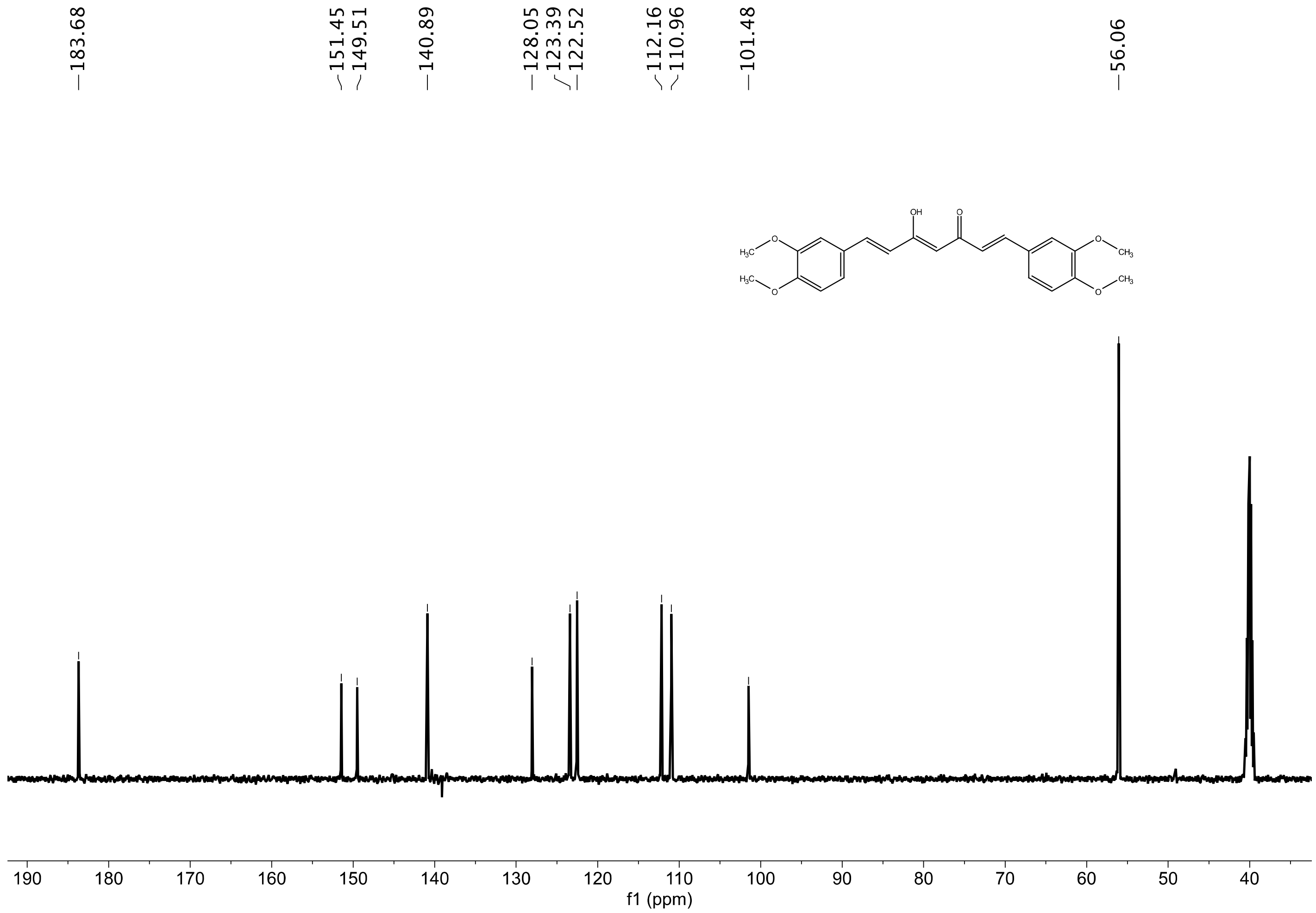


**Fig S6.** 13C NMR spectrum of DiMeOC (100 MHz, DMSO-$d_6$).

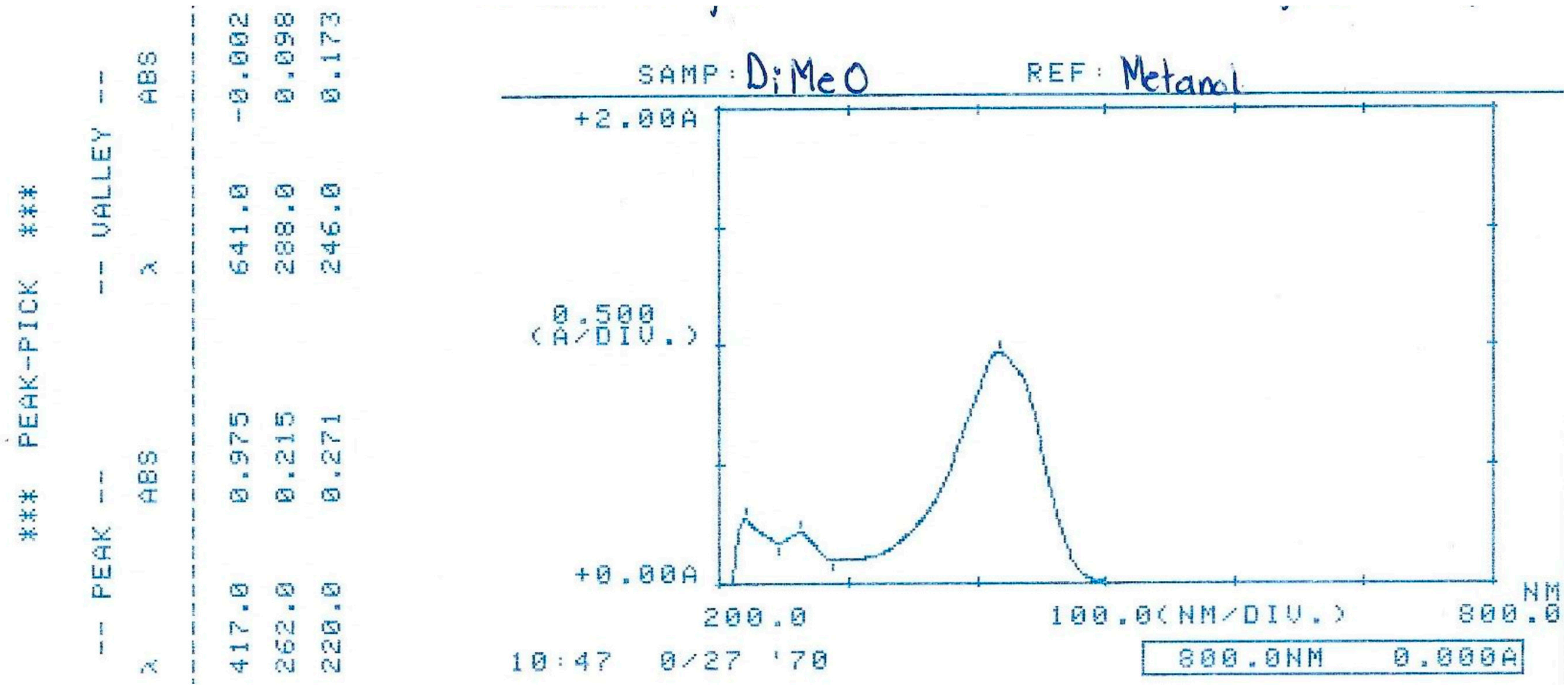


**Fig S7.** UV-VIS spectrum in methanol of DiMeOC.

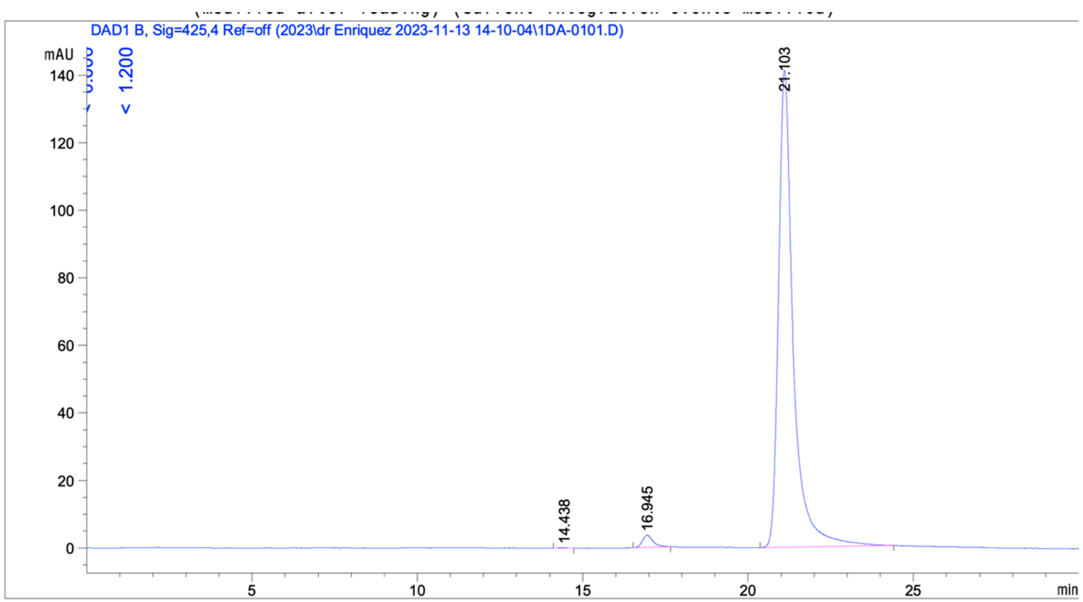


Signal 1: DAD1 B, Sig=425,4 Ref=off

| Peak # | RetTime [min] | Type | Width [min] | Area [mAU*s] | Height [mAU] | Area % |
|---|---|---|---|---|---|---|
| 1 | 14.438 | BB | 0.2067 | 3.60961 | 2.16300e-1 | 0.0812 |
| 2 | 16.945 | BB | 0.3063 | 82.44865 | 3.63930 | 1.8543 |
| 3 | 21.103 | BB | 0.4564 | 4360.39209 | 141.14056 | 98.0646 |

Totals : 4446.45035 144.99616

**Fig S8.** HPLC spectrum of DiMeOC (417nm, $CH_3CN/H_2O$ (0.02% formic acid) 55:45)

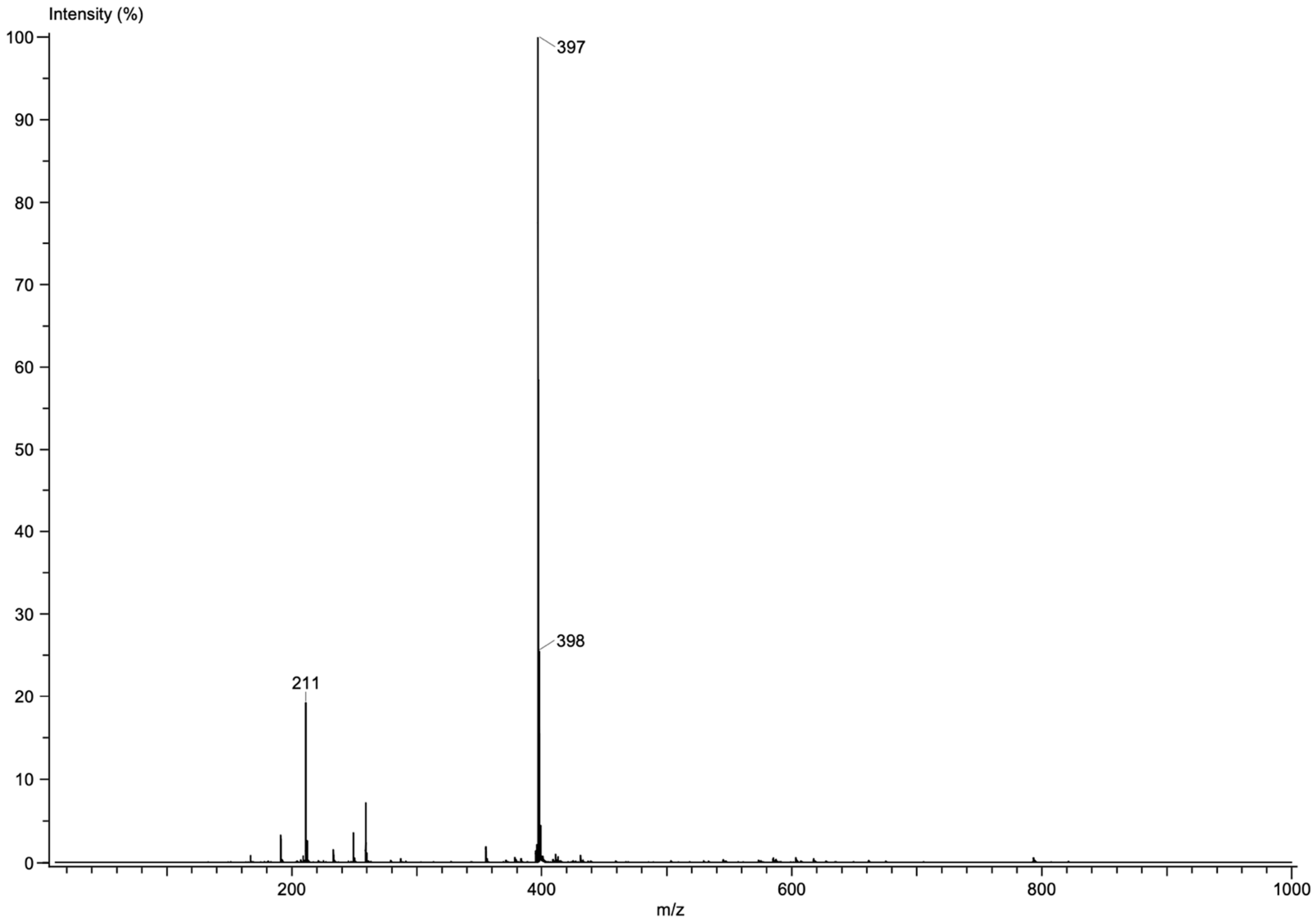


**Fig S9.** Mass Spectrum of DiMeOC (DART$^{+}$).

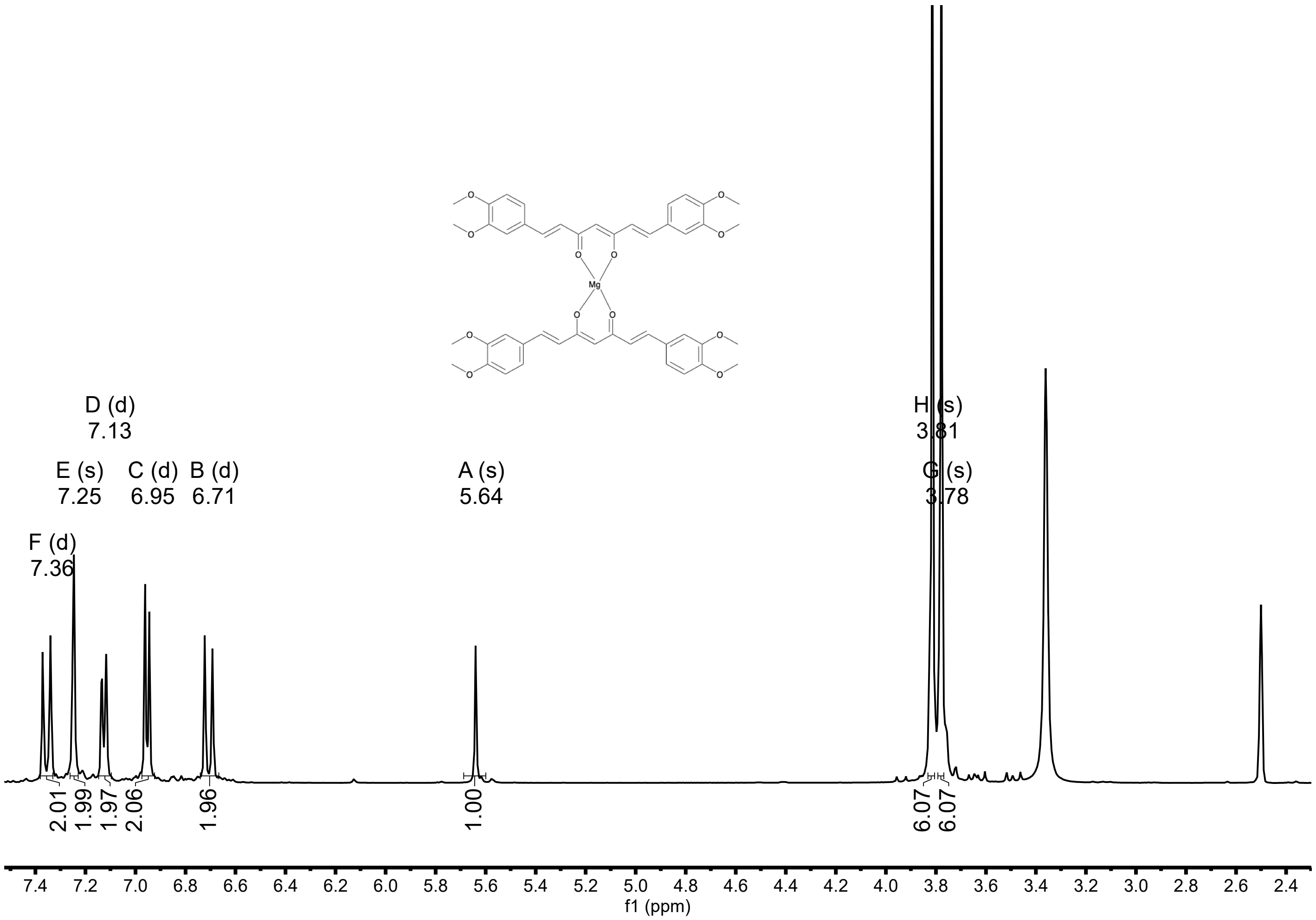


**Fig S10**. $^{1}$H NMR spectrum of DiMeOC-Mg (400 MHz, DMSO- $d_6$)

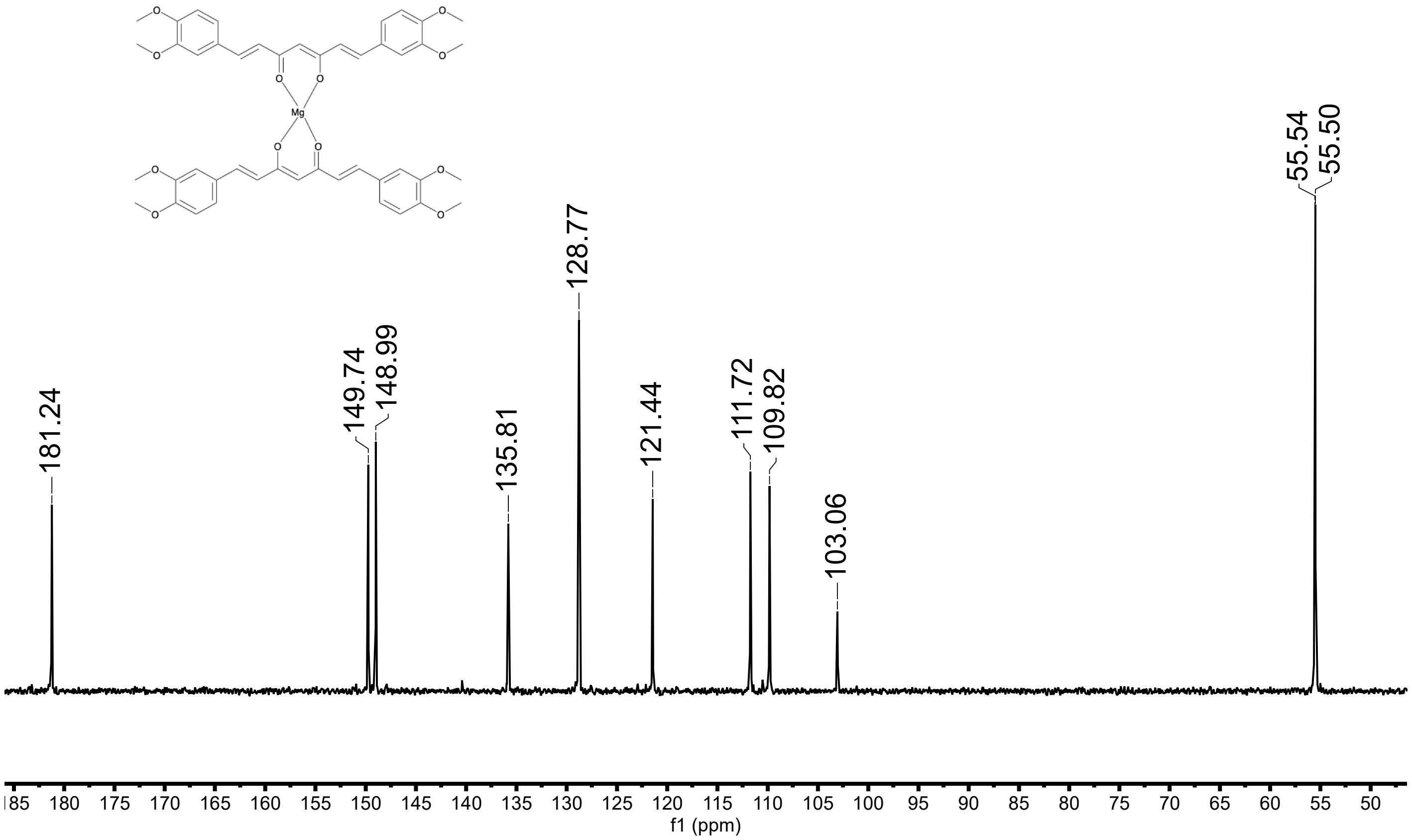


**Fig S11.** $^{13}$C NMR spectrum of DiMeOC-Mg (100 MHz, DMSO-$d_6$).

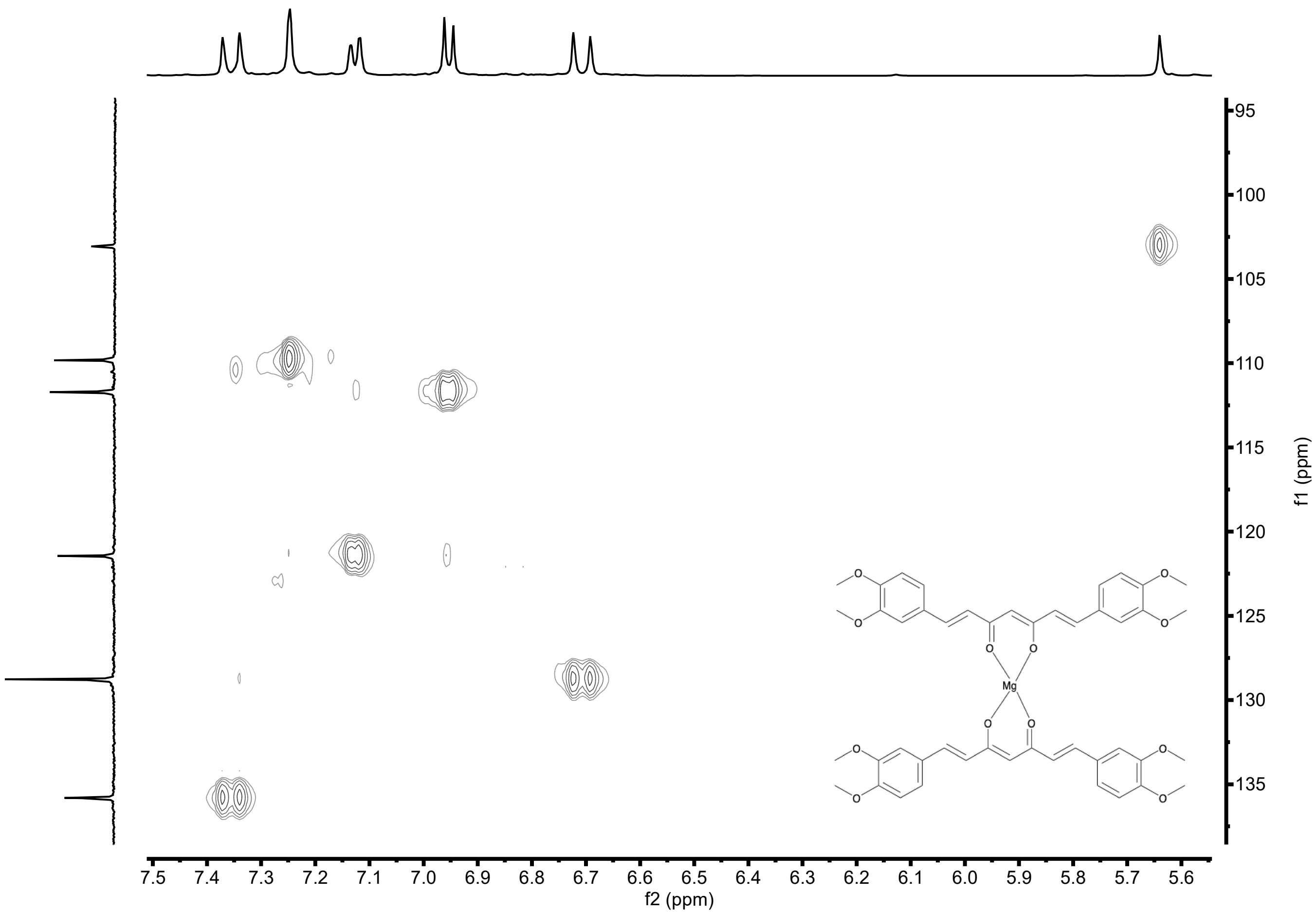


**Fig S12.** HSQC NMR spectrum of DiMeOC-Mg (400 MHz, DMSO-$d_6$).

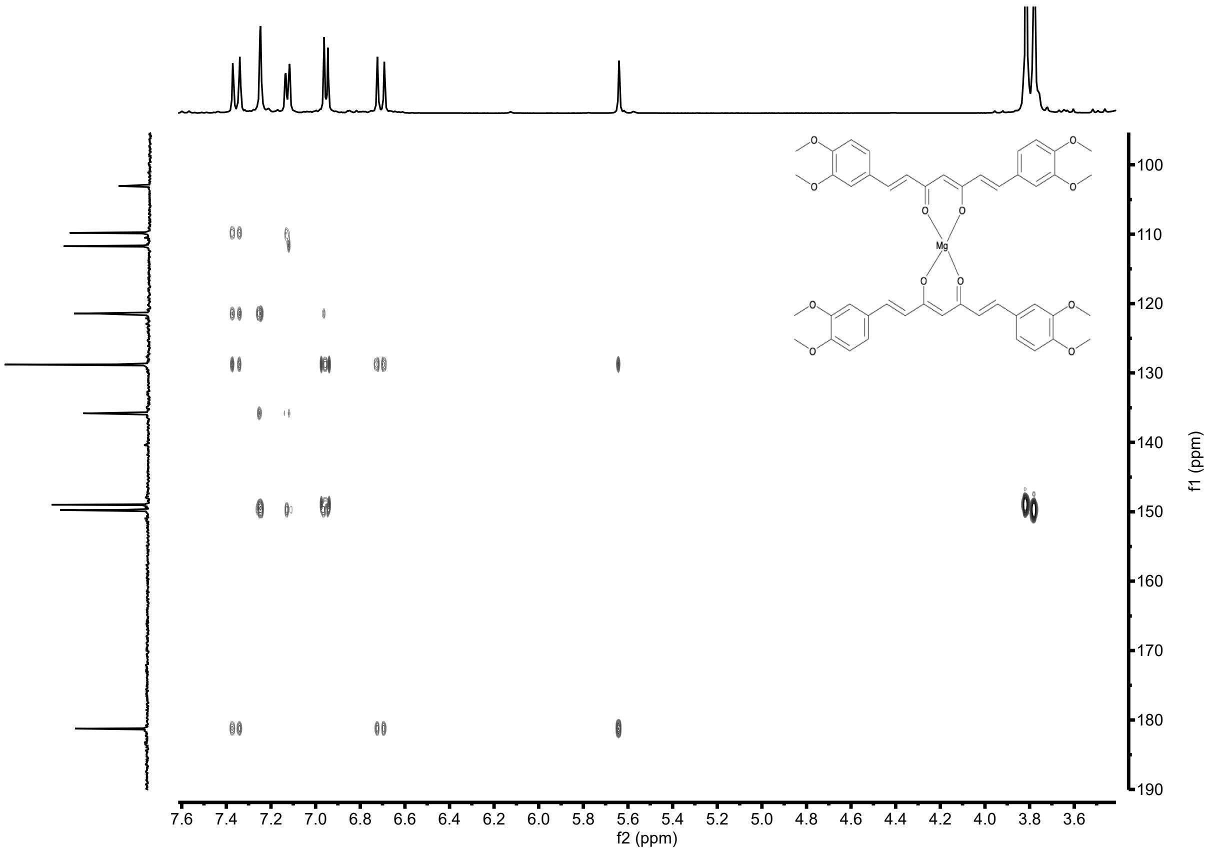


**Fig S13.** HMBC NMR spectrum of DiMeOC-Mg (400 MHz, DMSO-$d_6$).

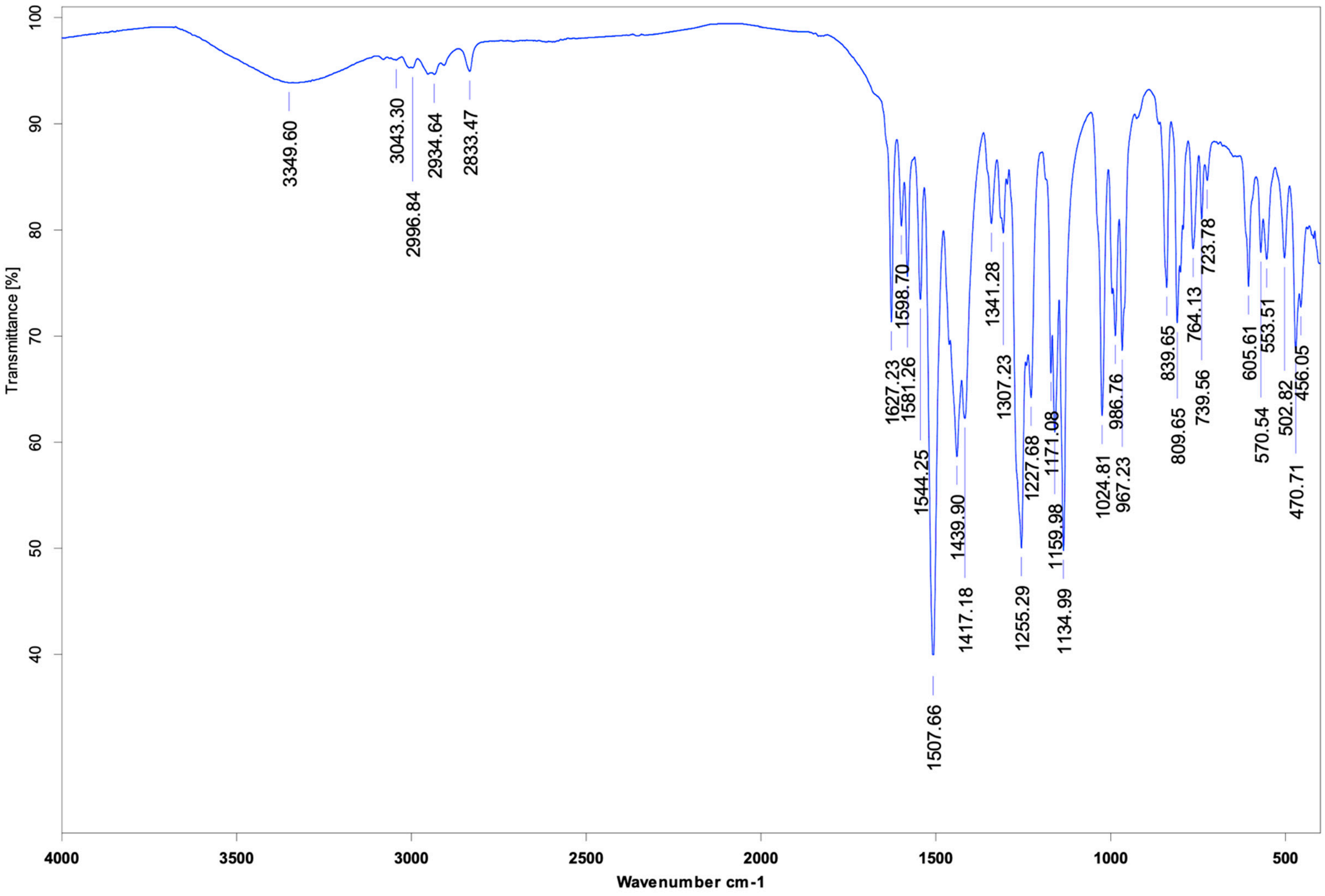


**Fig S14.** IR Spectrum of DiMeOC-Mg.

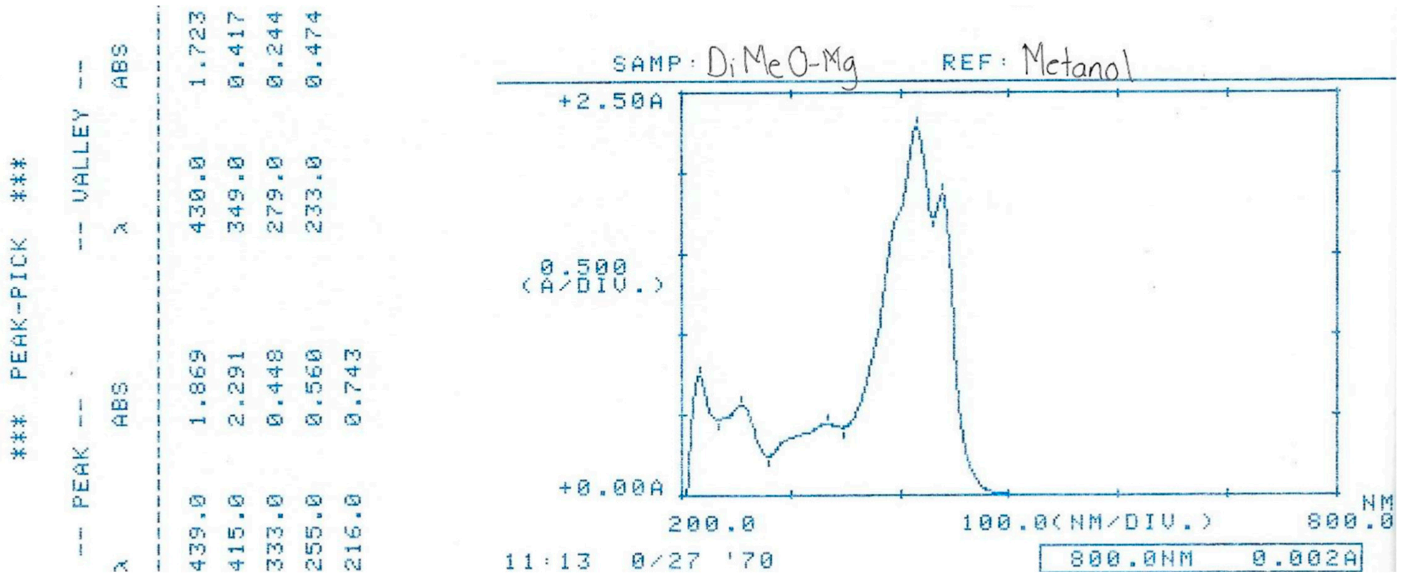


**Fig S15.** UV-VIS spectrum in methanol of DiMeOC-Mg .

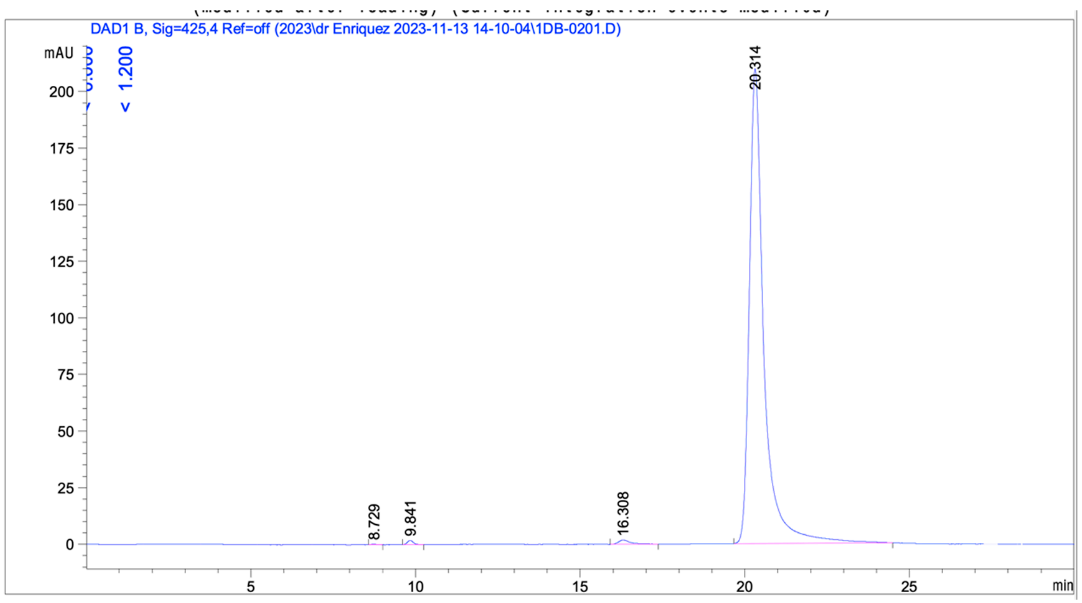


**Fig S16.** HPLC spectrum of DiMeOC-Mg (417 nm, $CH_3CN/H_2O$ (0.02% formic acid) 55:45).

Signal 1: DAD1 B, Sig=425,4 Ref=off

| Peak # | RetTime [min] | Type | Width [min] | Area [mAU*s] | Height [mAU] | Area % |
|---|---|---|---|---|---|---|
| 1 | 8.729 | BB | 0.1436 | 3.30270 | 3.01962e-1 | 0.0507 |
| 2 | 9.841 | BB | 0.1913 | 23.72007 | 1.90404 | 0.3642 |
| 3 | 16.308 | BV R | 0.3436 | 50.56177 | 1.91799 | 0.7763 |
| 4 | 20.314 | BB | 0.4502 | 6435.36230 | 209.61371 | 98.8088 |

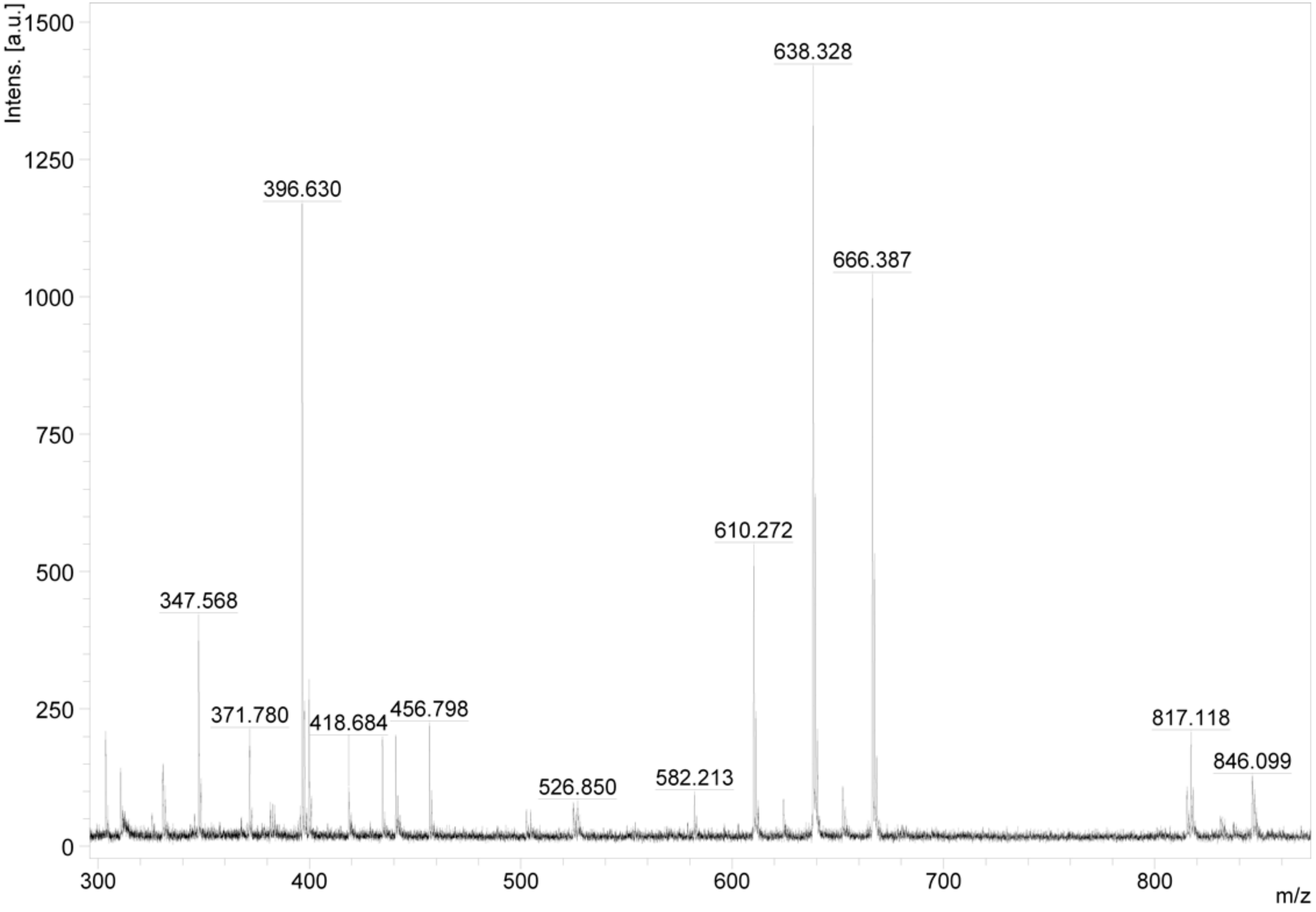


**Fig S17.** Mass Spectrum of DiMeOC-Mg (MALDI-TOF).

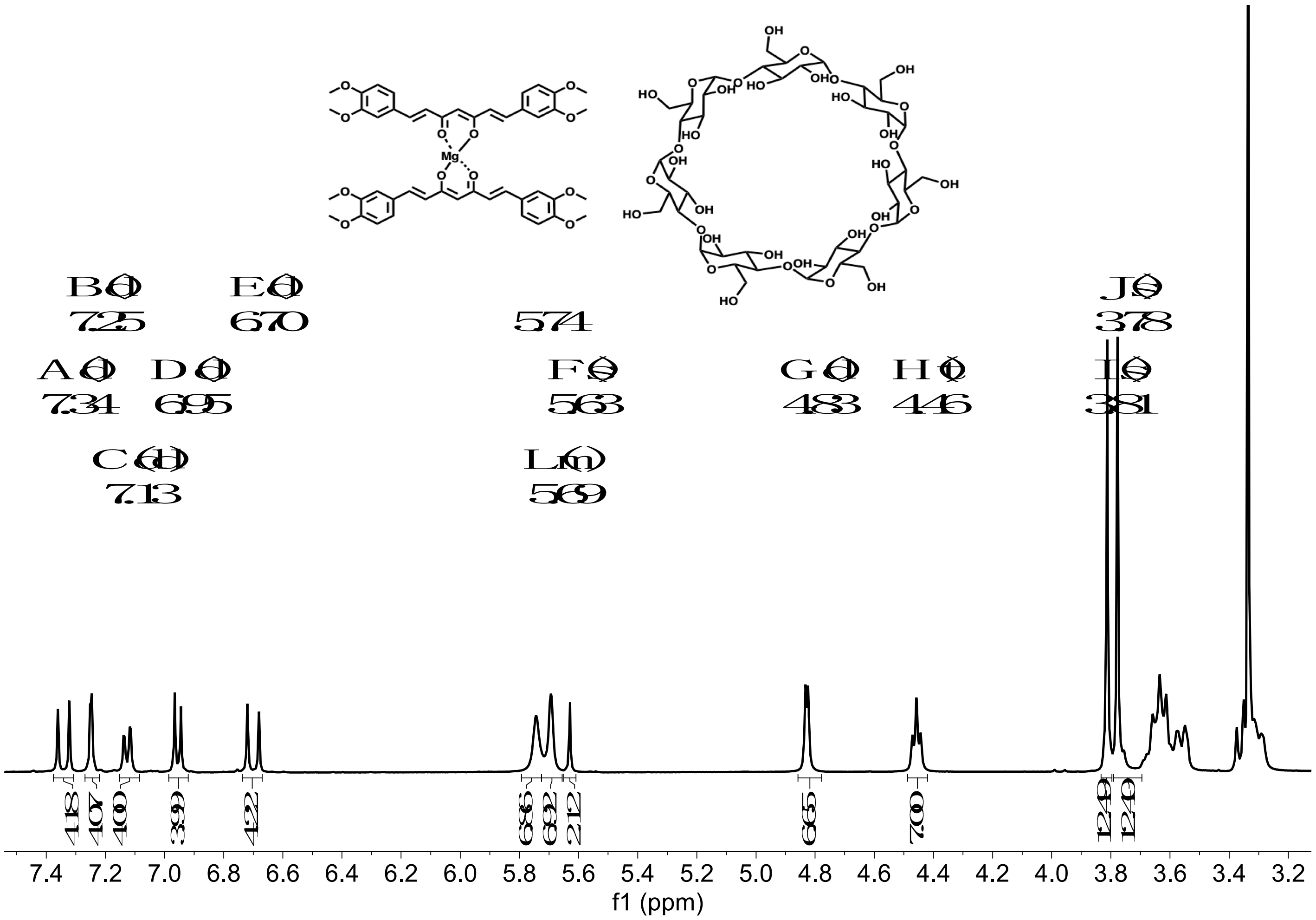


**Fig S18**. $^{1}H$ NMR spectrum of DiMeOC-Mg-BCD (400 MHZ, DMSO- $d_6$).

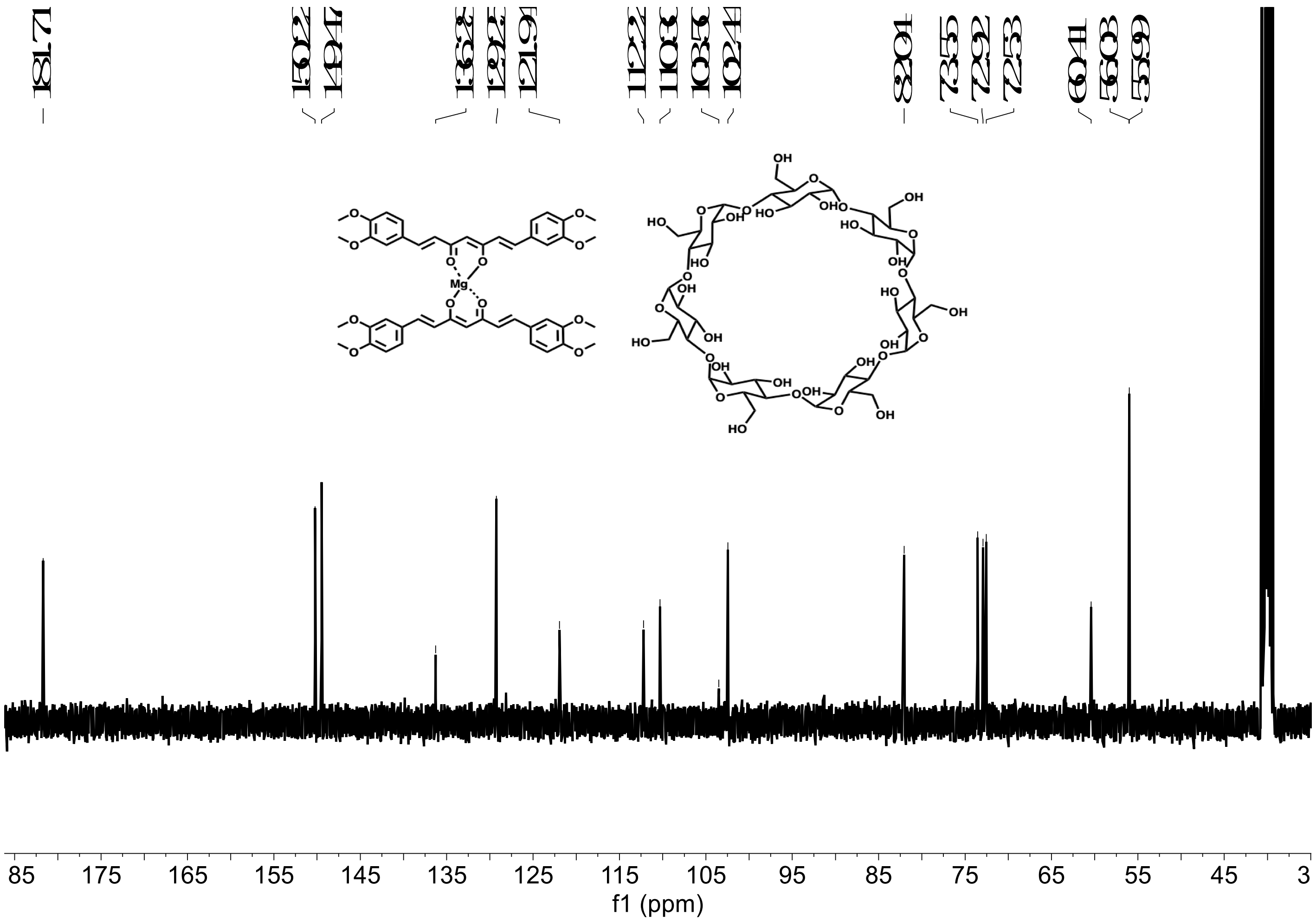


**Fig S19.** $^{13}$C NMR spectrum of DiMeOC-Mg-BCD (100 MHz, DMSO-$d_6$).

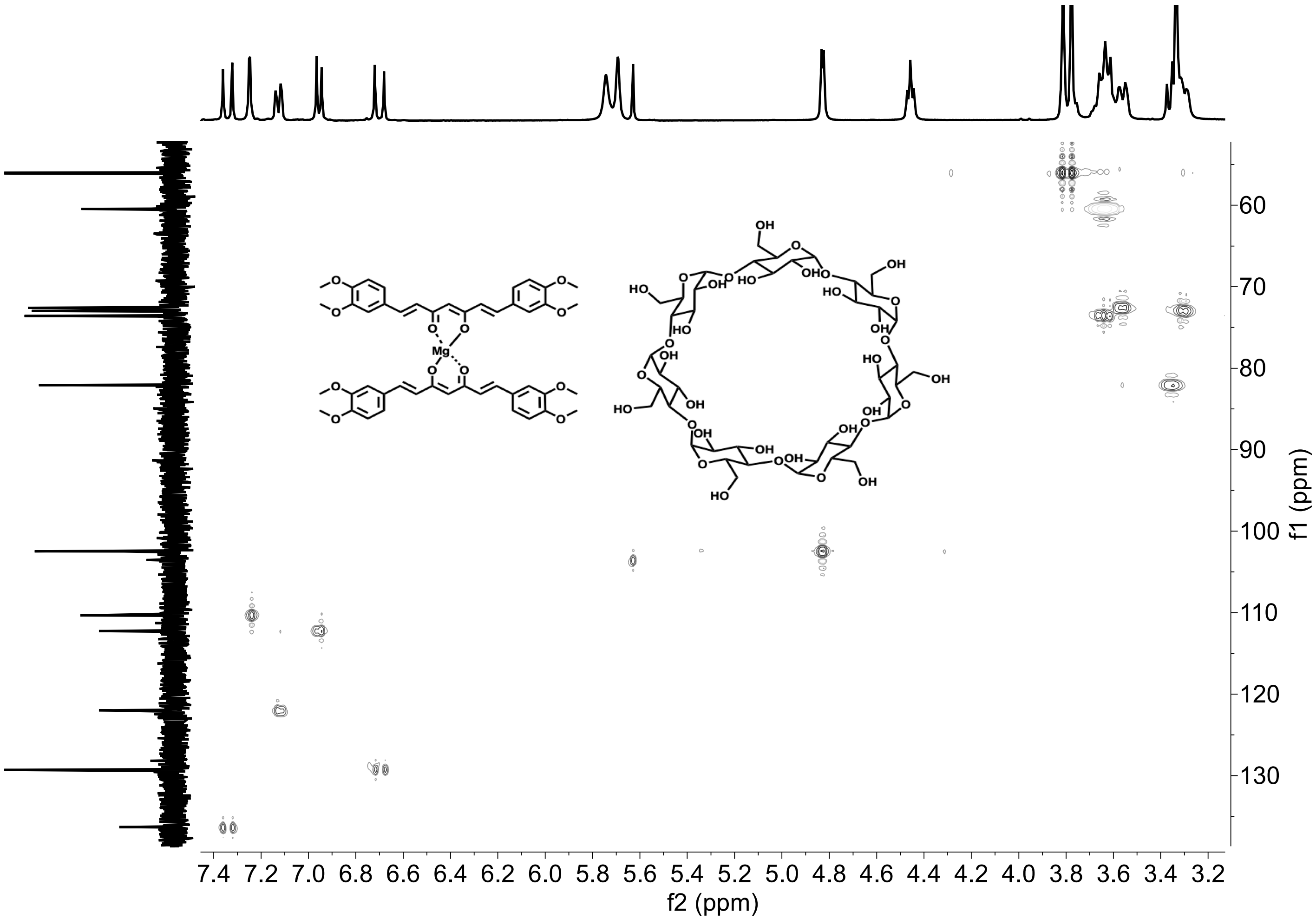


**Fig S20.** HSQC NMR spectrum of DiMeOC-Mg-BCD (400 MHz, DMSO-$d_6$).

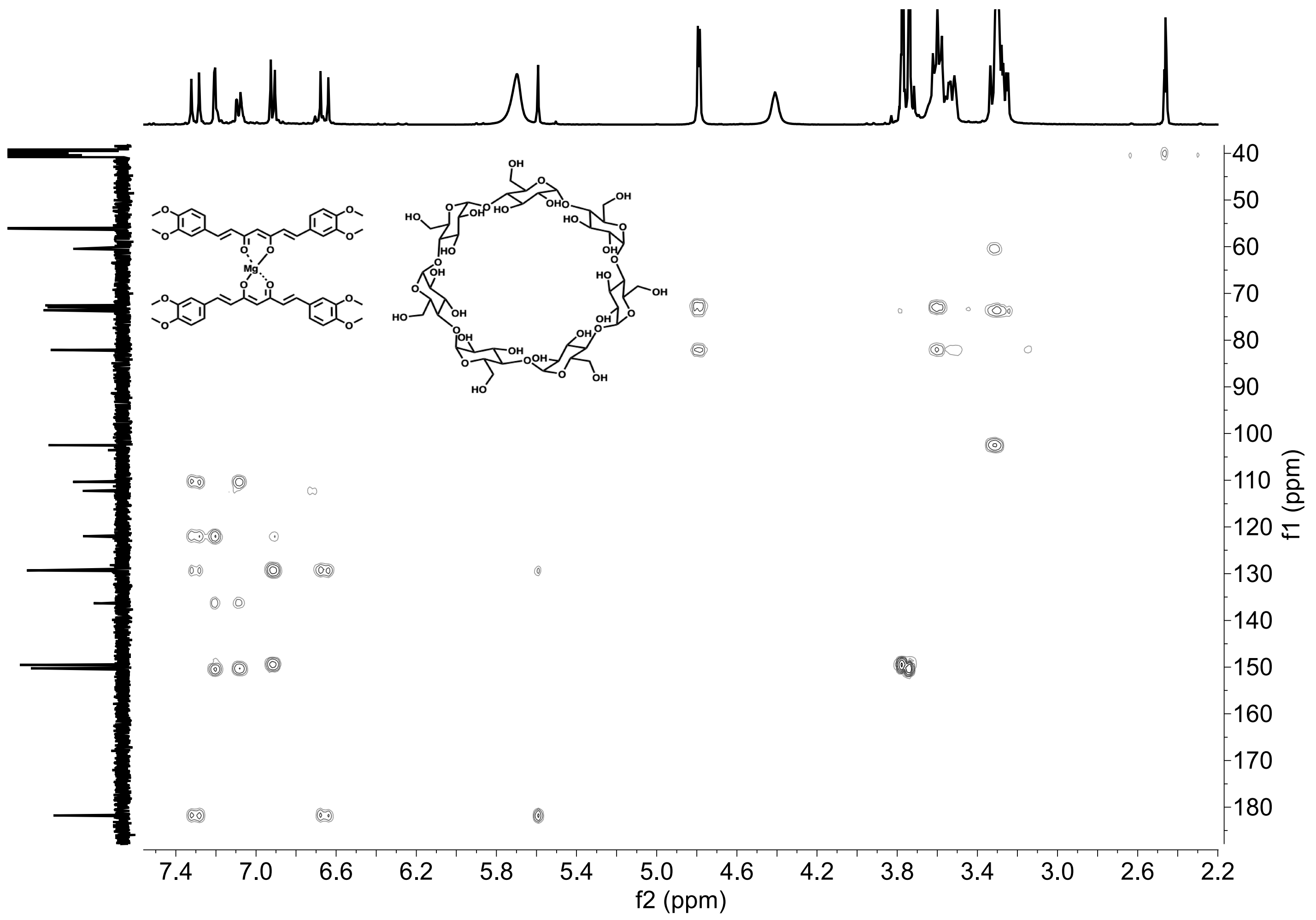


**Fig S21.** HMBC NMR spectrum of DiMeOC-Mg-BCD (400 MHz, DMSO-$d_6$).

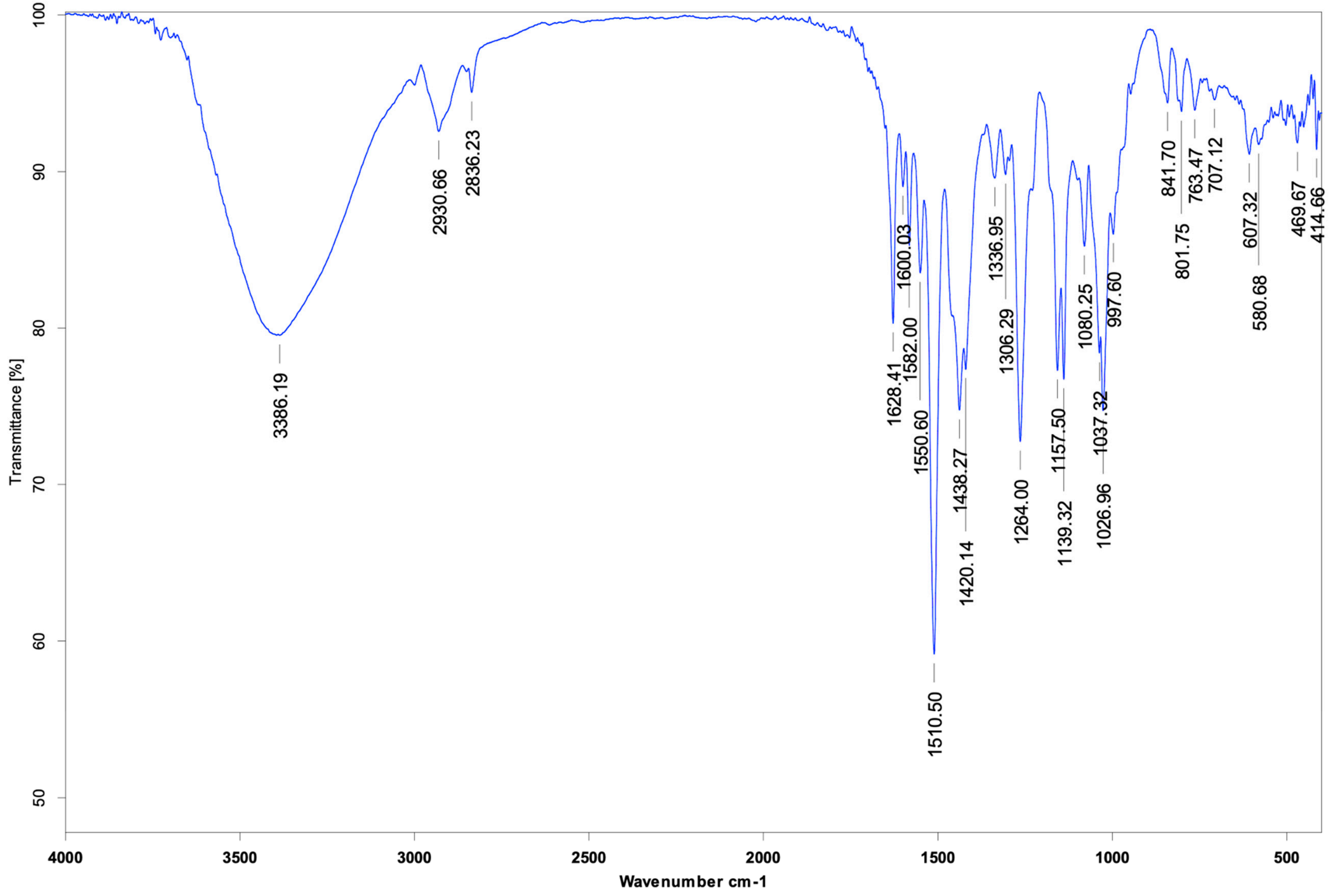


**Fig S22.** IR Spectrum of DiMeOC-Mg-BCD.

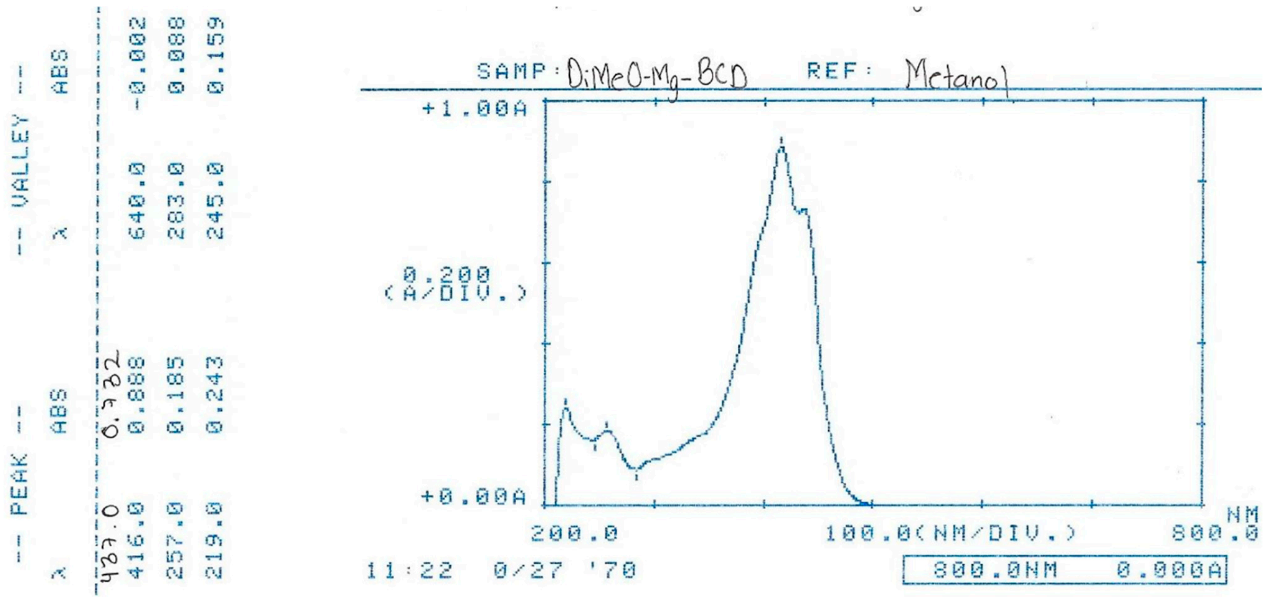


**Fig S23.** UV-VIS spectrum in methanol of DiMeOC-Mg-BCD .

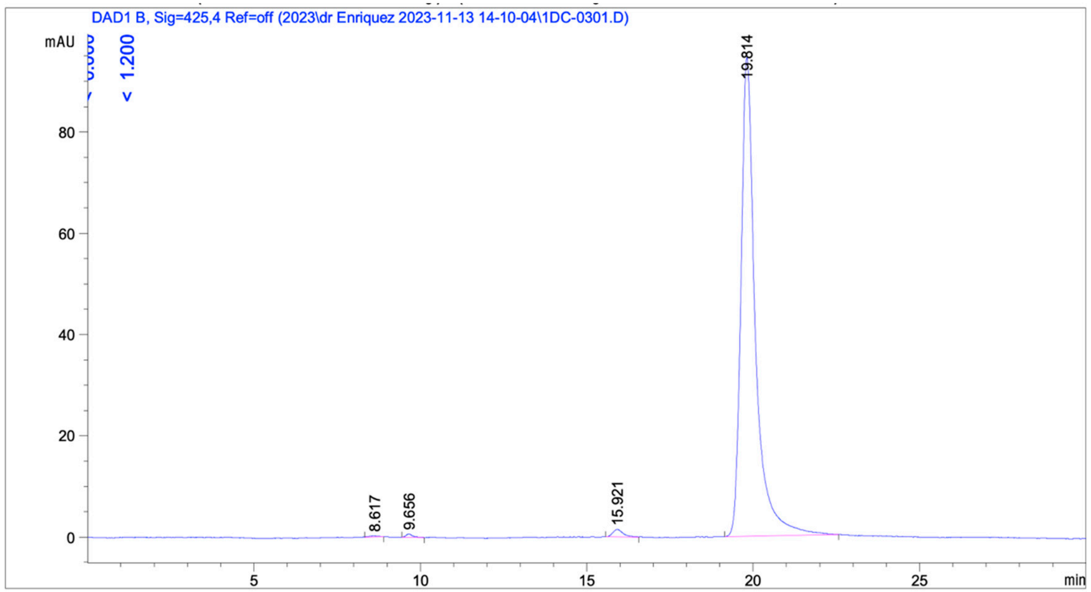


**Fig S24.** HPLC spectrum of DiMeOC-Mg-BCD (417nm, $CH_3CN/H_2O$ (0.02% formic acid) 55:45).

| Peak # | RetTime [min] | Type | Width [min] | Area [mAU*s] | Height [mAU] | Area % |
|---|---|---|---|---|---|---|
| 1 | 8.617 | BB | 0.1878 | 3.62682 | 2.37953e-1 | 0.1252 |
| 2 | 9.656 | BB | 0.1708 | 8.75976 | 6.51972e-1 | 0.3023 |
| 3 | 15.921 | BB | 0.2585 | 31.41060 | 1.49874 | 1.0841 |
| 4 | 19.814 | BB | 0.4463 | 2853.59155 | 94.51001 | 98.4884 |

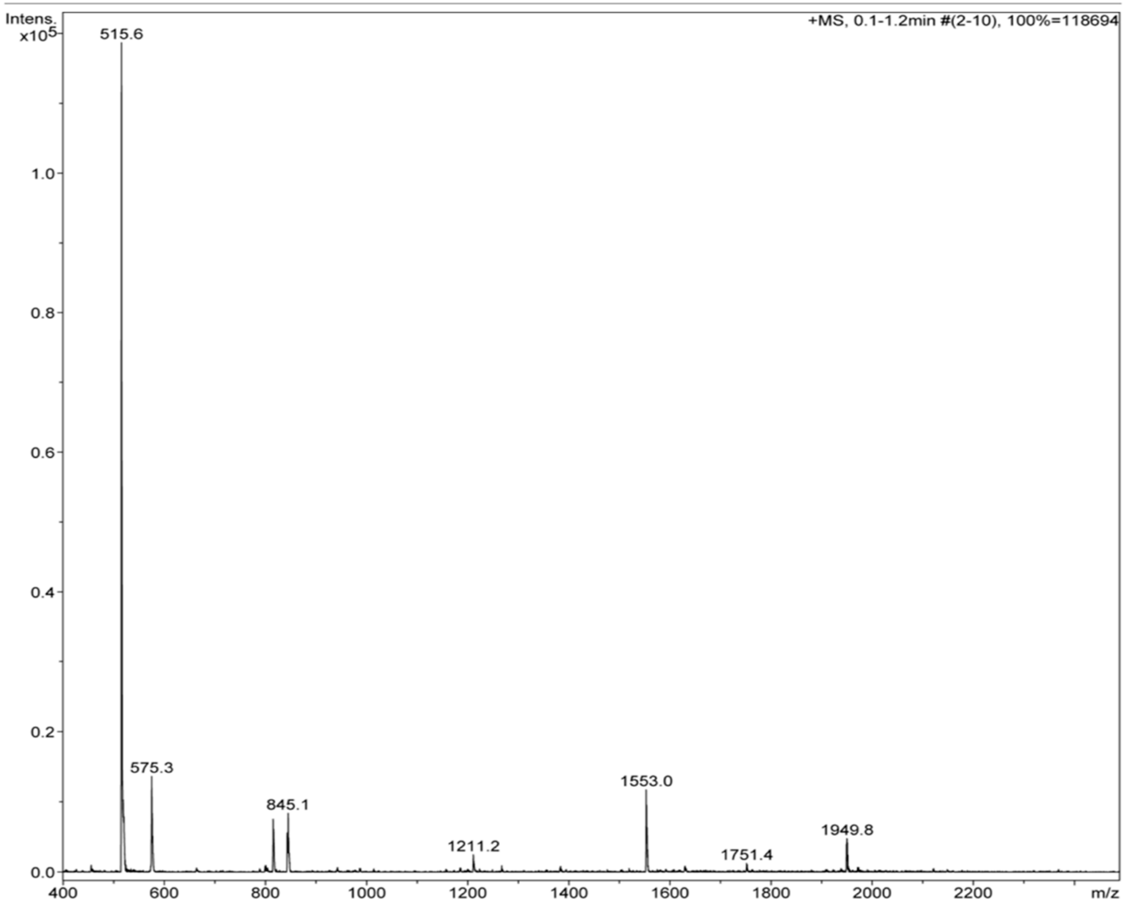


**Fig S25.** Mass Spectrum of DiMeOC-Mg-BCD (ESI).

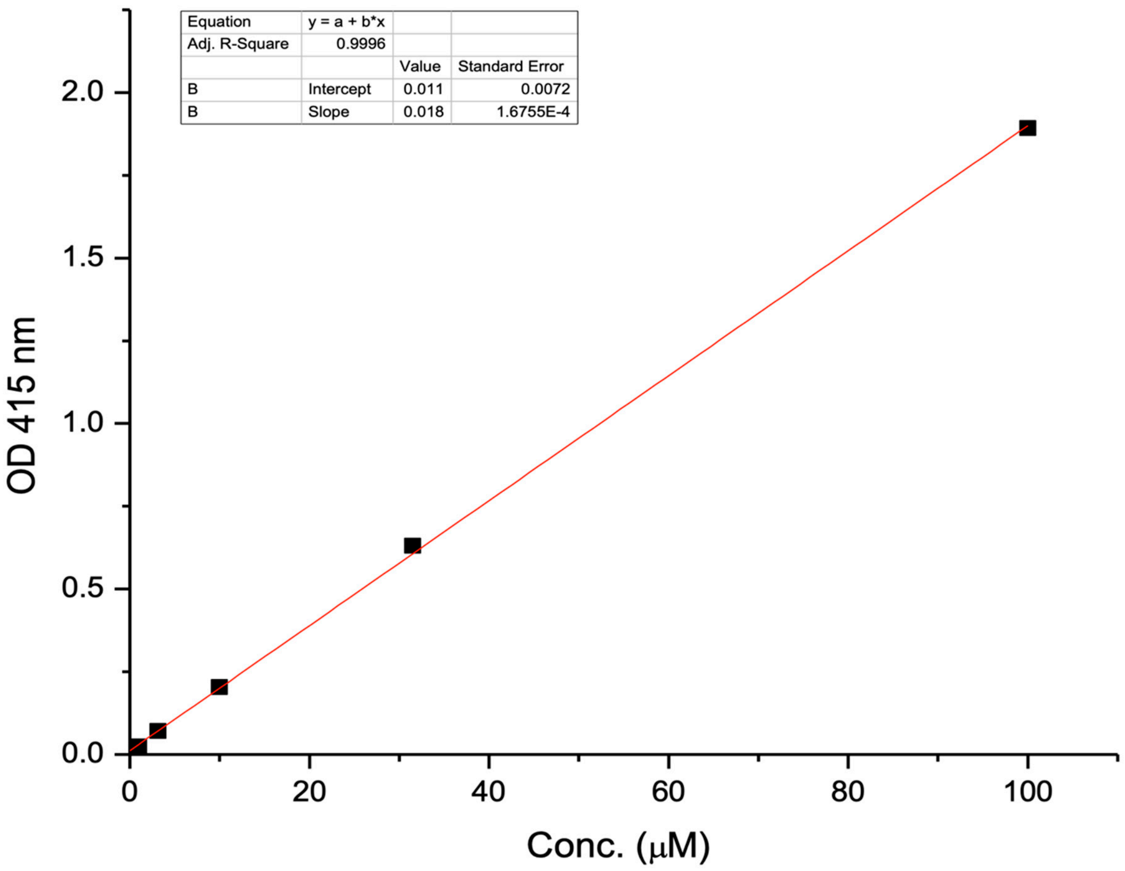


**Fig S26.** Standard curve of DiMeOC-Mg (415nm, 1-Octanol).

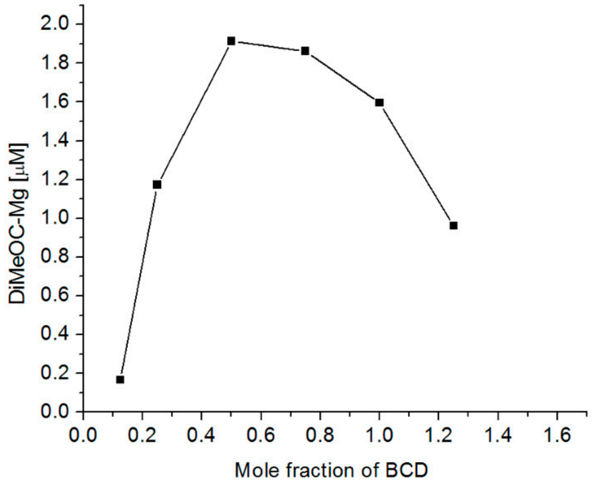


**Fig S27.** Inclusion ratio DiMeOC-Mg and BCD (PBS media).

The inclusion ratio test results between DiMeOC-Mg and BCD are shown in Fig. s27. The mole fraction represents r of BCD in the mixture. when r = 0.5, the molar concentration value of DiMeOC-Mg was the largest, concluding that the inclusion complex was formed to the molar ratio of 1:1.

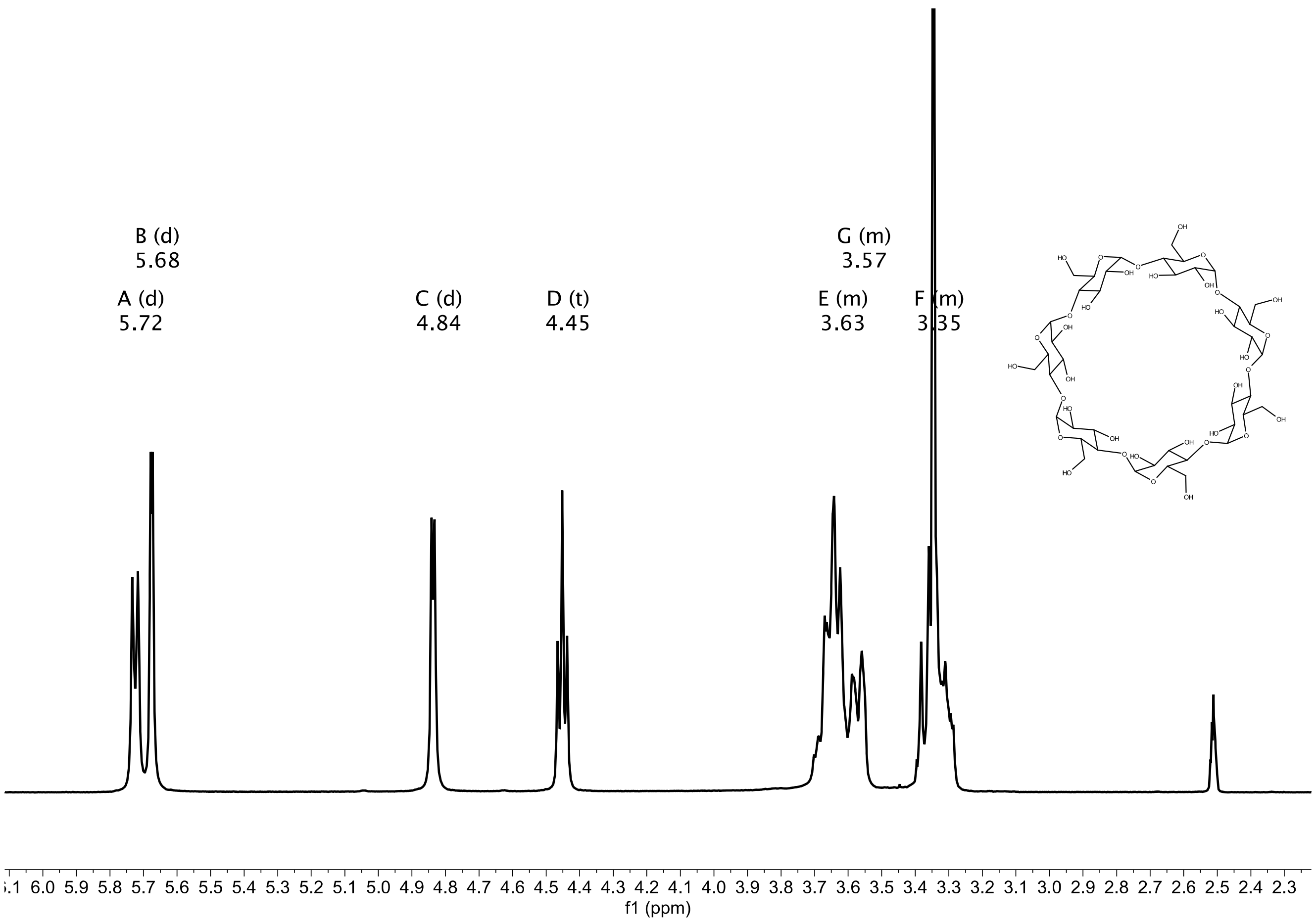


**Fig S28.** $^{1}$H NMR spectrum of Beta-cyclodextrin (BCD, 400 MHz, DMSO- $d_6$).

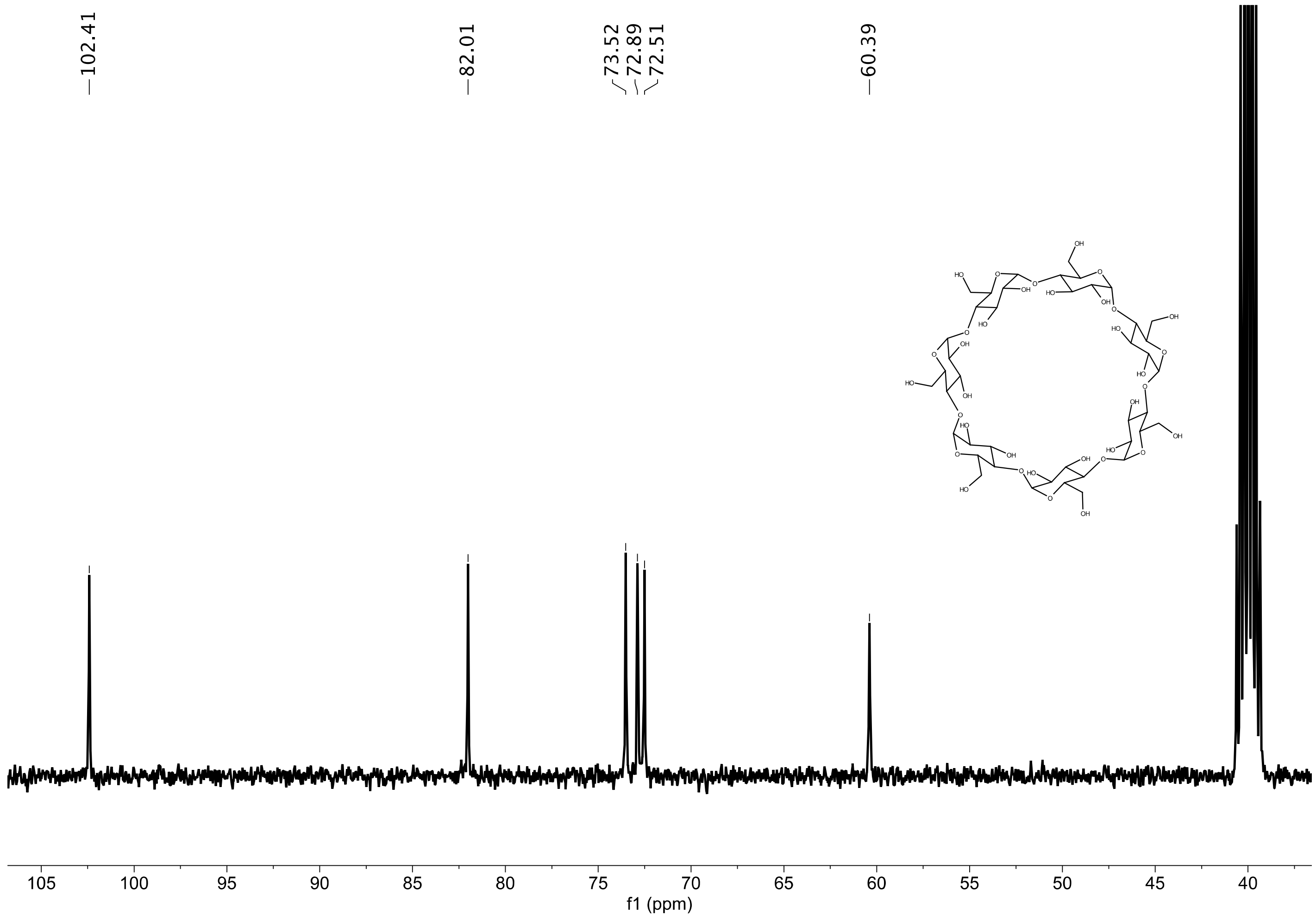


**Fig S29.** $^{13}C$ NMR spectrum of BCD (100 MHz, DMSO-$d_6$).

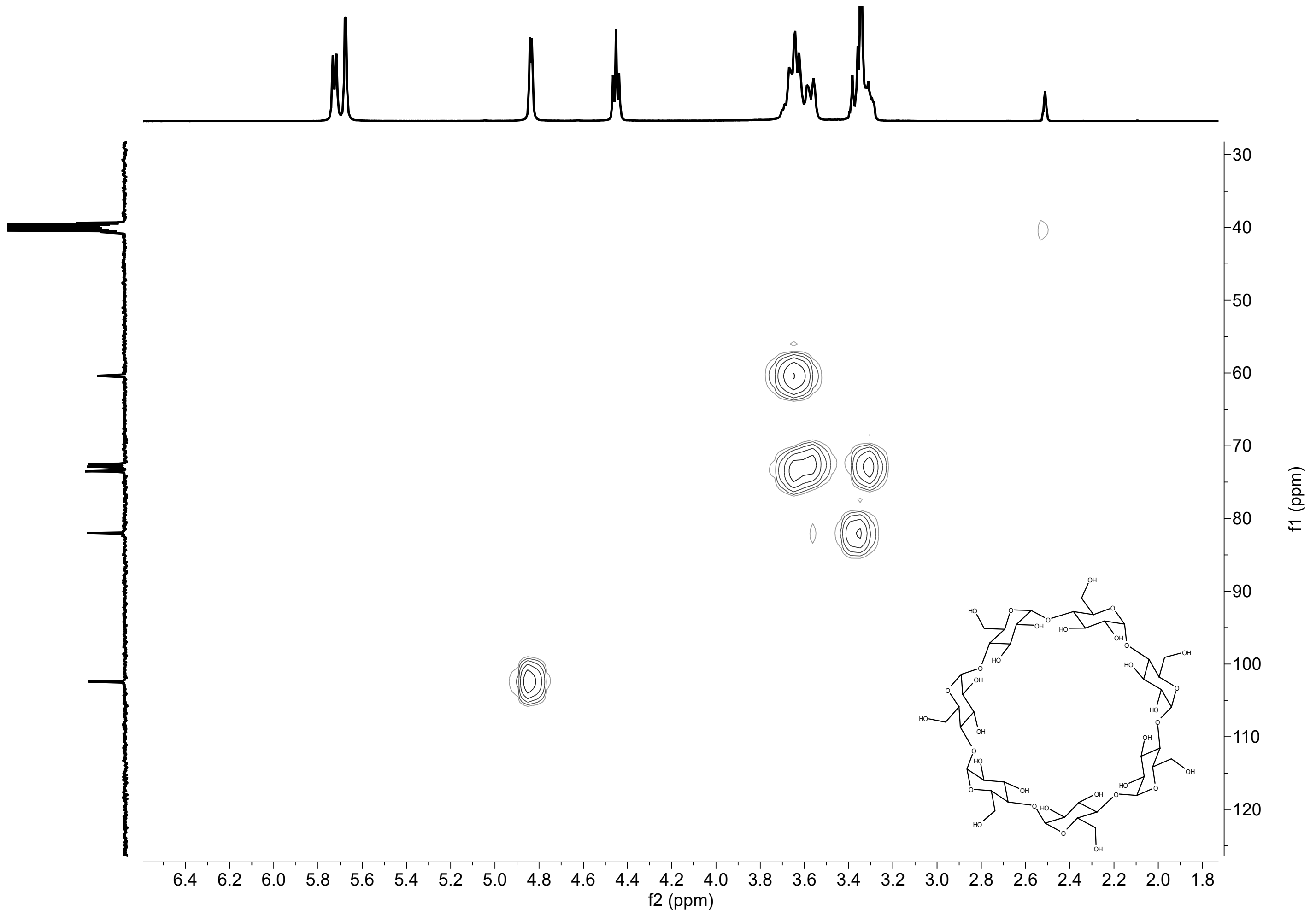


**Fig S30.** HSQC NMR spectrum of BCD (500 MHz, DMSO-$d_6$).

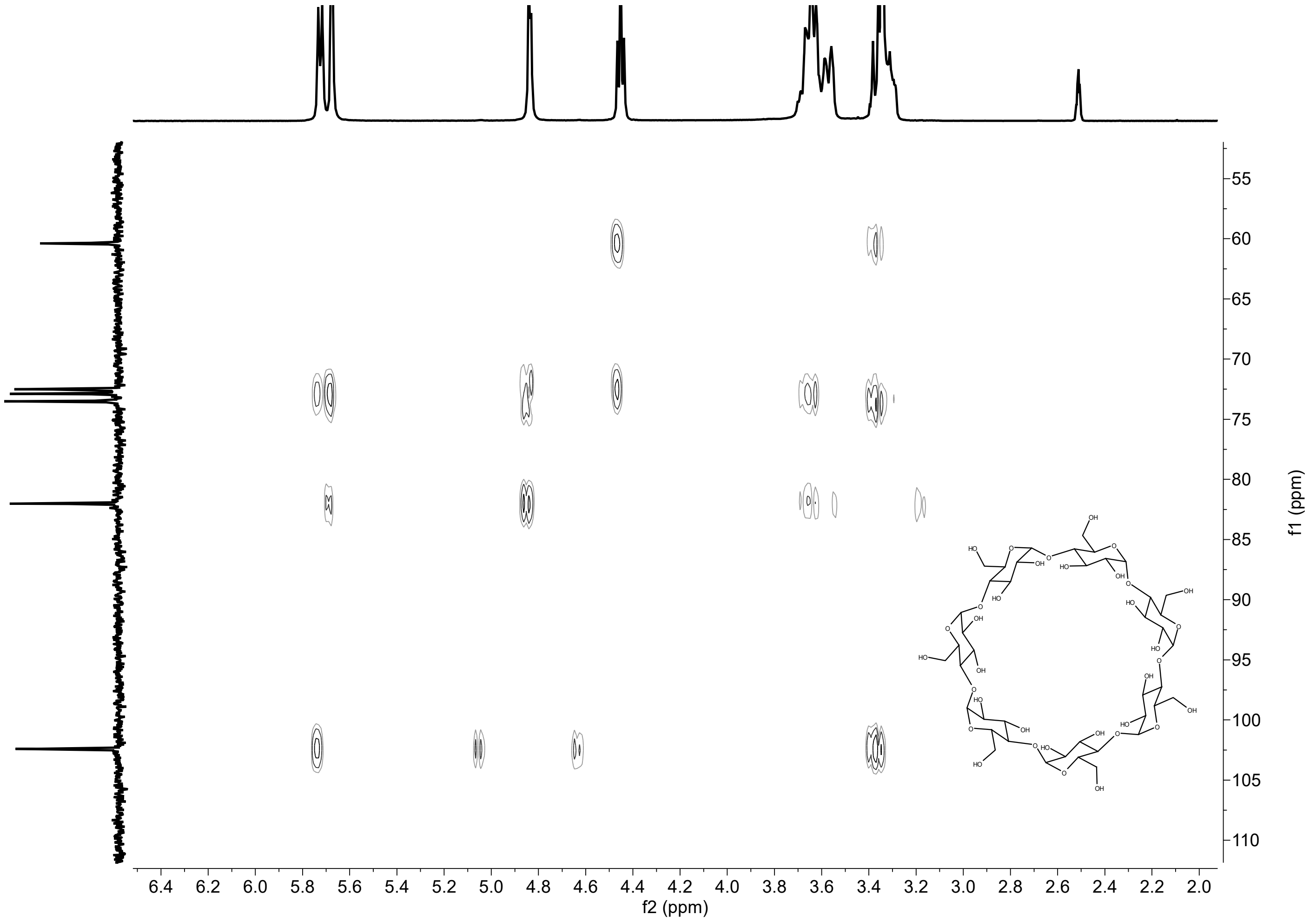


**Fig S31.** HMBC NMR spectrum of BCD (500 MHz, DMSO-$d_6$).

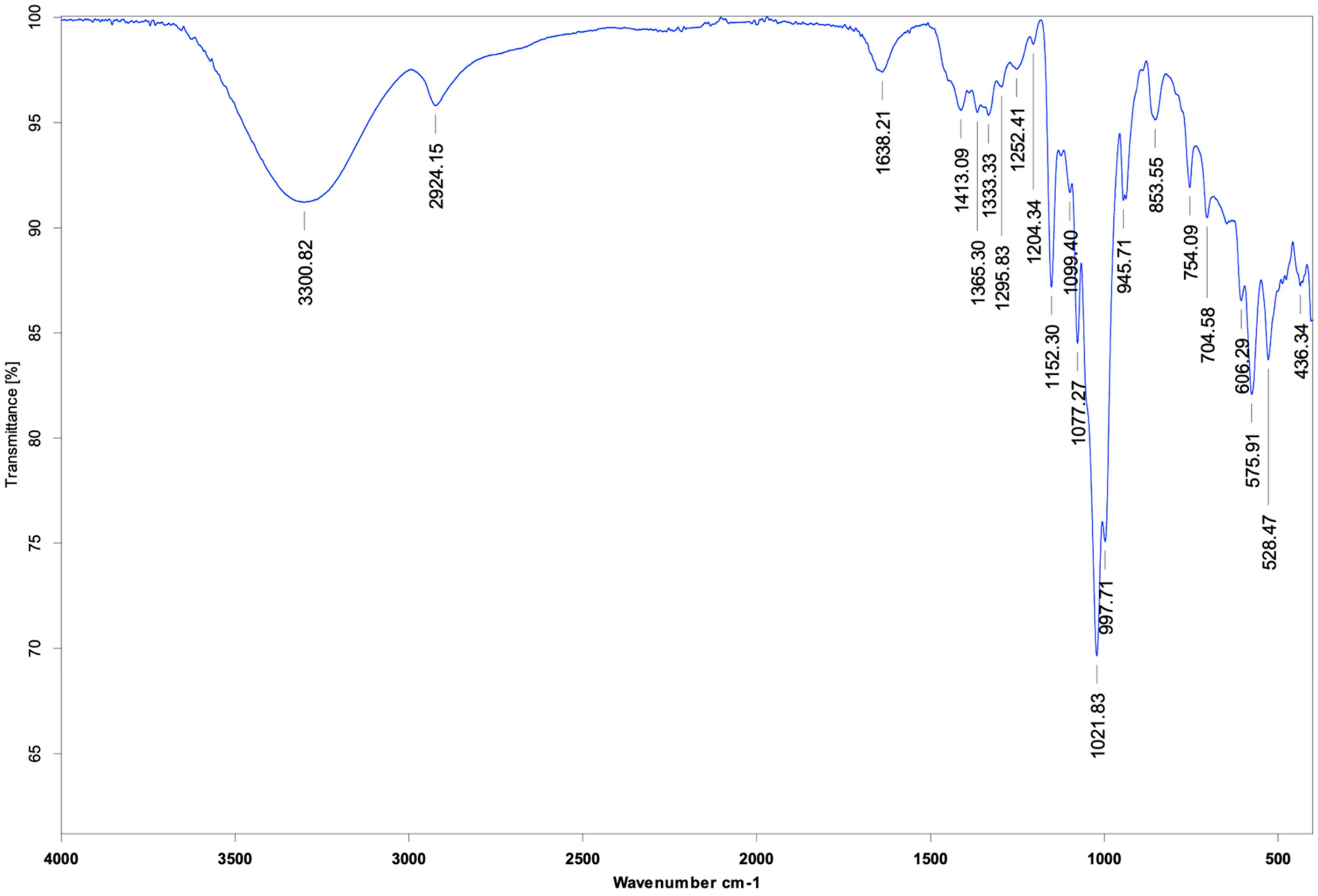


**Fig S32.** IR Spectrum of BCD.
